# Antimicrobial and Biodegradable 3D Printed Scaffolds for Orthopedic Infections

**DOI:** 10.1021/acsbiomaterials.3c00115

**Published:** 2023-06-20

**Authors:** Anshu Dubey, Henri Vahabi, Vignesh Kumaravel

**Affiliations:** † International Centre for Research on Innovative Biobased Materials (ICRI-BioM)—International Research Agenda, Lodz University of Technology Żeromskiego 116, Lodz 90-924, Poland; ‡ Université de Lorraine, CentraleSupélec, LMOPS, F-57000 Metz, France

**Keywords:** Infectious diseases, Orthopedic Surgery, Bone defects, Bioprinting, Biocompatible Materials

## Abstract

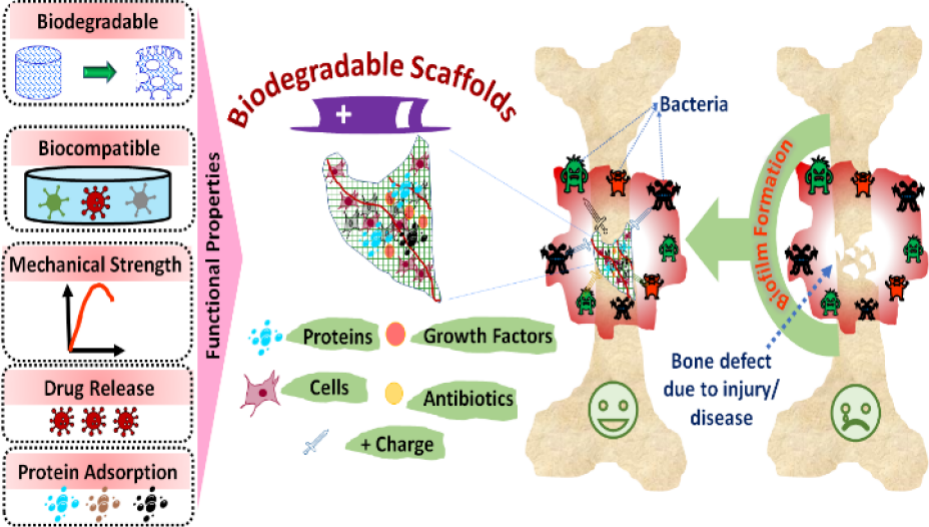

In bone tissue engineering, the performance of scaffolds underpins the success of the healing of bone. Microbial infection is the most challenging issue for orthopedists. The application of scaffolds for healing bone defects is prone to microbial infection. To address this challenge, scaffolds with a desirable shape and significant mechanical, physical, and biological characteristics are crucial. 3D printing of antibacterial scaffolds with suitable mechanical strength and excellent biocompatibility is an appealing strategy to surmount issues of microbial infection. The spectacular progress in developing antimicrobial scaffolds, along with beneficial mechanical and biological properties, has sparked further research for possible clinical applications. Herein, the significance of antibacterial scaffolds designed by 3D, 4D, and 5D printing technologies for bone tissue engineering is critically investigated. Materials such as antibiotics, polymers, peptides, graphene, metals/ceramics/glass, and antibacterial coatings are used to impart the antimicrobial features for the 3D scaffolds. Polymeric or metallic biodegradable and antibacterial 3D-printed scaffolds in orthopedics disclose exceptional mechanical and degradation behavior, biocompatibility, osteogenesis, and long-term antibacterial efficiency. The commercialization aspect of antibacterial 3D-printed scaffolds and technical challenges are also discussed briefly. Finally, the discussion on the unmet demands and prevailing challenges for ideal scaffold materials for fighting against bone infections is included along with a highlight of emerging strategies in this field.

## 1 Introduction

Bone is a natural composite made of organic (collagen) and inorganic (hydroxyapatite) substances with a hierarchal structural configuration with exceptional self-healing characteristics and minor damages. Despite such a capability, bone defects are the undesirable catastrophes that dominate the bone repair capability. Bone defects due to trauma, diseases, cancer, and poor prognosis are the major problems for orthopedic patients in clinical practice.^1^ Moreover, treating infections along with bone defects is another intractable challenge in orthopedics. The standard methods for restoring bone defects are autografts and xenografts.^2,3^ However, bone infections pose a risk for bone transplantation surgeries. Despite successes *in vitro*, there are failures during *in vivo* experiments because of the severe infection leading to revision surgery. Therefore, infections are life-threatening for patients and burden society economically. The typical cause of bone infection is the adhesion and proliferation of pathogens of the genus of *Staphylococcus*, specifically *Staphylococcus aureus*.^4,5^ The resistance mechanism of the *Staphylococcus aureus* strain, which is present in the skin, makes it more vulnerable and unassailable to an opportunistic pathogen. The biofilm formation is more problematic because it provides a conducive environment through various mechanisms that facilitate long-term survival. First, the biofilms create an impenetrable and physical obstacle to antibiotics and immune cells, preventing the actions of macrophage phagocytosis, reactive oxygen, and antibodies secreted through immune host system cells (immune cells evoke a plethora of stresses such as acid stress, proteases, nitrogen stress, antimicrobial peptides, nutritional immunity, and reactive oxygen to eradicate invading pathogens). Second, due to phenotypic diversity, bacteria living in biofilm make it resistant to antibiotic treatment. Besides, these biofilm bacteria can endure comparatively higher antibiotic doses without damage to the films making them more resistant to drugs.^6^ Moreover, the surrounding bone tissues suffer severe damage, giving rise to bone resorption through bacteria virulence factors.^7,8^

**Figure 1:**
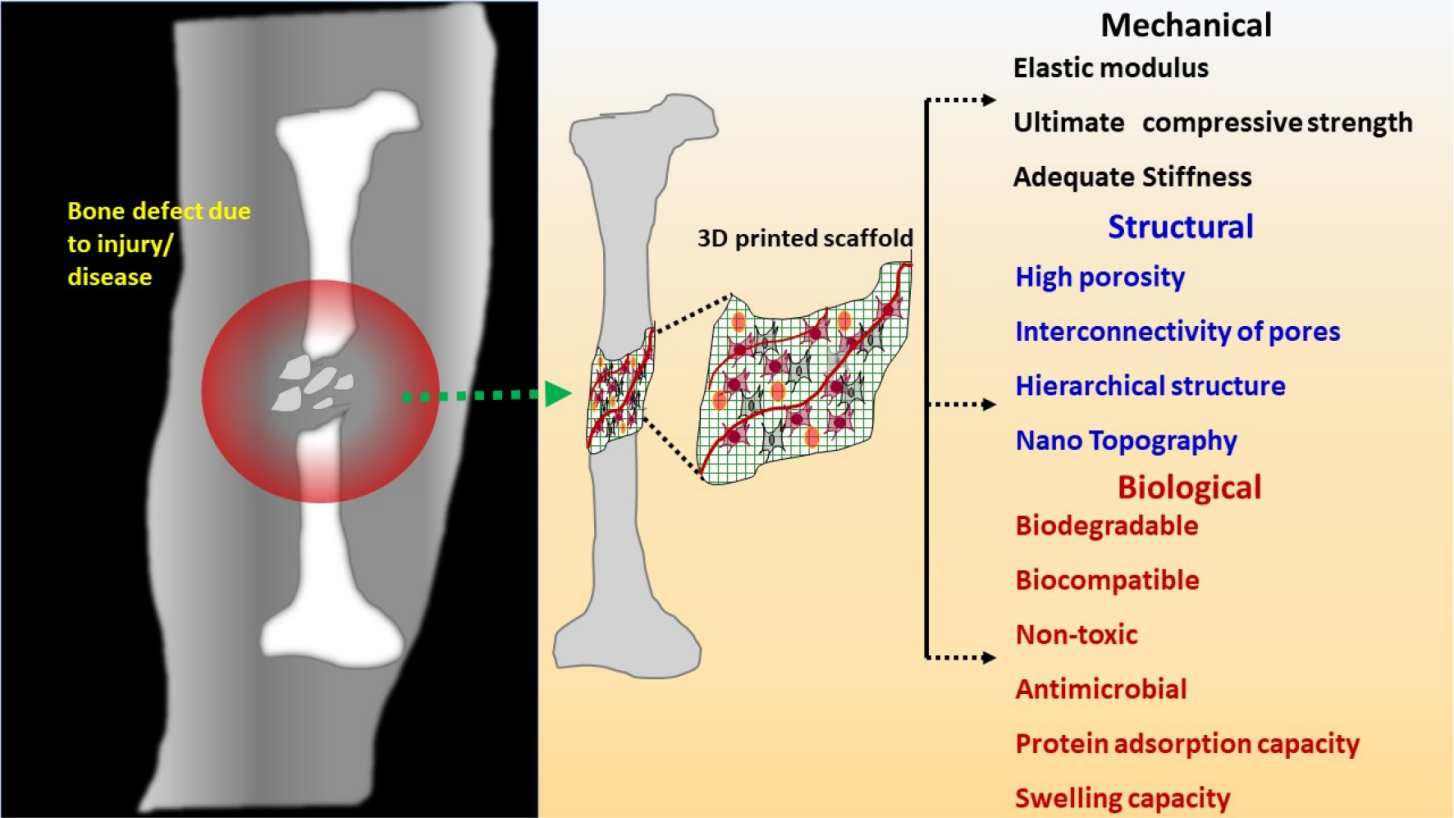
Schematic showing the major factors that need consideration during the design of antibacterial and biodegradable scaffolds to support bone regeneration. Given the complex loading condition on bones, the designed scaffolds should have adequate stiffness and compressive strength to prove their candidacy for load-bearing application. The structural integrity of biodegradable scaffolds depends on the amount of porosity and the size of interconnected pores in the construct that need critical thinking while deciding the designing parameters. The functional properties of scaffolds (antimicrobial, biocompatibility, and protein adsorption) can be enhanced by accommodating the various bioactive elements in the constructs.

Bone tissue engineering can be used to develop therapeutic strategies for curing bone abnormalities. Three-dimensional (3D) porous scaffolds are often used to treat bone defects and guide new bone regeneration. An ideal 3D porous scaffold should hold significant characteristics, such as mechanical strength, biodegradability, optimum porosity, biocompatibility, and ability to load cells and growth factors. It provides a 3D environment for the different types of cells and growth factors immersed in scaffolds, stimulating bone tissue growth and leading to osseointegration.^9^ In particular, these scaffolds are designed to fight against infection, so incorporating antimicrobial materials into these scaffolds will be more promising.^10^ The significant properties of an ideal 3D-printed porous scaffold for bone tissue engineering are shown in Figure 1. For instance, the mechanical properties, drug loading concentration, and drug release rate are quite dependent on the pore size and amount of porosity in the fabricated 3D scaffolds.

The essential properties can be controlled by using the appropriate manufacturing techniques for 3D scaffolds. The conventional fabrication methods cannot fulfill the required design of an ideal 3D porous scaffold. Ideally, the designed scaffolds should support the complex loading condition inside the body and respond to the surrounding stimuli, such as chemical or biological signals. However, it is challenging to precisely control the pore size, geometry, and spatial distribution of interconnected pores via conventional fabrication routes such as freeze-drying, particulate leaching, and solvent casting methods.

As depicted in Figure 1, the connectivity and pore size of the scaffold can influence cell growth and bone regeneration. Scaffolds with interconnected pores favor the transport of nutrients, penetration of cells, cell migration, formation of new bone tissues, and vascularization. For the mineralization of bone, a minimum pore size of ∼100 μm is required for any kind of scaffold. Studies have also revealed that scaffolds with pore sizes in the range of 250–600 μm could facilitate cell proliferation, extracellular matrix production, capillary formation, and new bone formation.^2,11^

Thus, there is a need for advanced fabrication methods to overcome the limitations that exist in conventional approaches. Interestingly, additive manufacturing (AM) is the breakthrough for fabricating complex biomimetic 3D scaffolds.^12,13^ AM of 3D interconnected porous scaffolds is the most reproducible technique as it fabricates computer-controlled 3D porous structures of biomaterials. The various available AM methods are fused deposition modeling (FDM),^14^ selective laser sintering (SLS), inkjet printing, extrusion-based printing and stereolithography.^12,15^ 3D-printing techniques offer to fabricate the 3D porous structures of various biomaterials such as ceramics, polymers, metals, and composites with well-defined and customized shapes and structures that mimic the 3D structure of human bone tissues.

The major challenge in the AM technique is the selection of appropriate biomaterials so that the smooth transfer of clinical translation can occur. Based on the requirement, the opted biomaterials must have adequate mechanical strength, biodegradability, biocompatibility, and bioactivity *in vivo*. Based on the degradation characteristics of the materials, implant materials can be categorized into nondegradable and temporary scaffolds. Nondegradable scaffolds are made of titanium and its alloys, stainless steel, or cobalt chromium, which exhibit outstanding mechanical features with cytocompatibility. Despite that, there are some limitations to the performance of nondegradable implants when used for temporary purposes, such as (i) stress-shielding effect, (ii) release of some toxic ions, (iii) smooth surface of the implants is favorable for the adhesion of bacteria, and (iv) resurgery is required to remove the implants after bone healing.^16^ The need of the hour is the synthesis of novel biomaterials that can overcome the above limitations. Thus, biodegradable materials (natural or synthetic) have grabbed the attention of orthopedic researchers. The biodegradable nature of materials offers to degrade in the body’s fluid environment with the process of bone healing and, eventually, eliminate the process of additional surgery after bone healing.^17^

Thus, the development and design of biodegradable 3D porous scaffolds for curing defects and infections have become the forefront of bone tissue engineering. Most recent reports cover only the significance of antimicrobial scaffolds for oral and skin tissue engineering. Curiously, none of them has explicitly focused on the advanced 3D-printing techniques to fabricate antimicrobial biodegradable scaffold bone tissue engineering. Considering the critical situation of surgical infections in bone tissue engineering equated with escalated healthcare costs, the scientific community has been actively involved in developing suitable and cost-effective antibacterial scaffolds for the last ten years. In addition to various biodegradable polymers, magnesium (Mg) and zinc (Zn)-based biodegradable metallic scaffolds are new choices of today’s researchers for healing minor bone defects. Researchers are exploring the therapeutic effect of these temporary metallic scaffolds for treating bone defects due to infections. A detailed elaboration on the different types of biodegradable antimicrobial 3D porous scaffolds and their unique characteristics for healing the infected bone, along with the manufacturing techniques, is needed, which is missing in the current literature. Further, the prospects of 4D and 5D printing have not yet been covered in the literature. Therefore, it is anticipated that a critical and highly focused evaluation of biodegradable 3D porous scaffolds via advanced 3D printing technology would appeal to new researchers in orthopedics.

Herein, the potential breakthroughs of state-of-the-art 3D porous biodegradable scaffolds for bone tissue engineering are critically discussed. Further, the insightful advantage of 3D printing for fabricating ideal biodegradable scaffold has been covered extensively. The importance of antibacterial strategies and other properties, such as protein adsorption and swelling, are also discussed in detail. Furthermore, the prospects of 4D and 5D printing are also disclosed. Lastly, the challenges, perspectives, and critical factors governing the success of biodegradable 3D porous scaffolds for orthopedics are also been briefed. Therefore, these key findings provide in-depth knowledge for developing antibacterial, biodegradable 3D scaffolds through innovative technology, focusing on the exciting state-of-the-art directions of 3D printing for future growth.

## 2 AM Techniques Available for 3D Printing of Scaffolds

**Figure 2:**
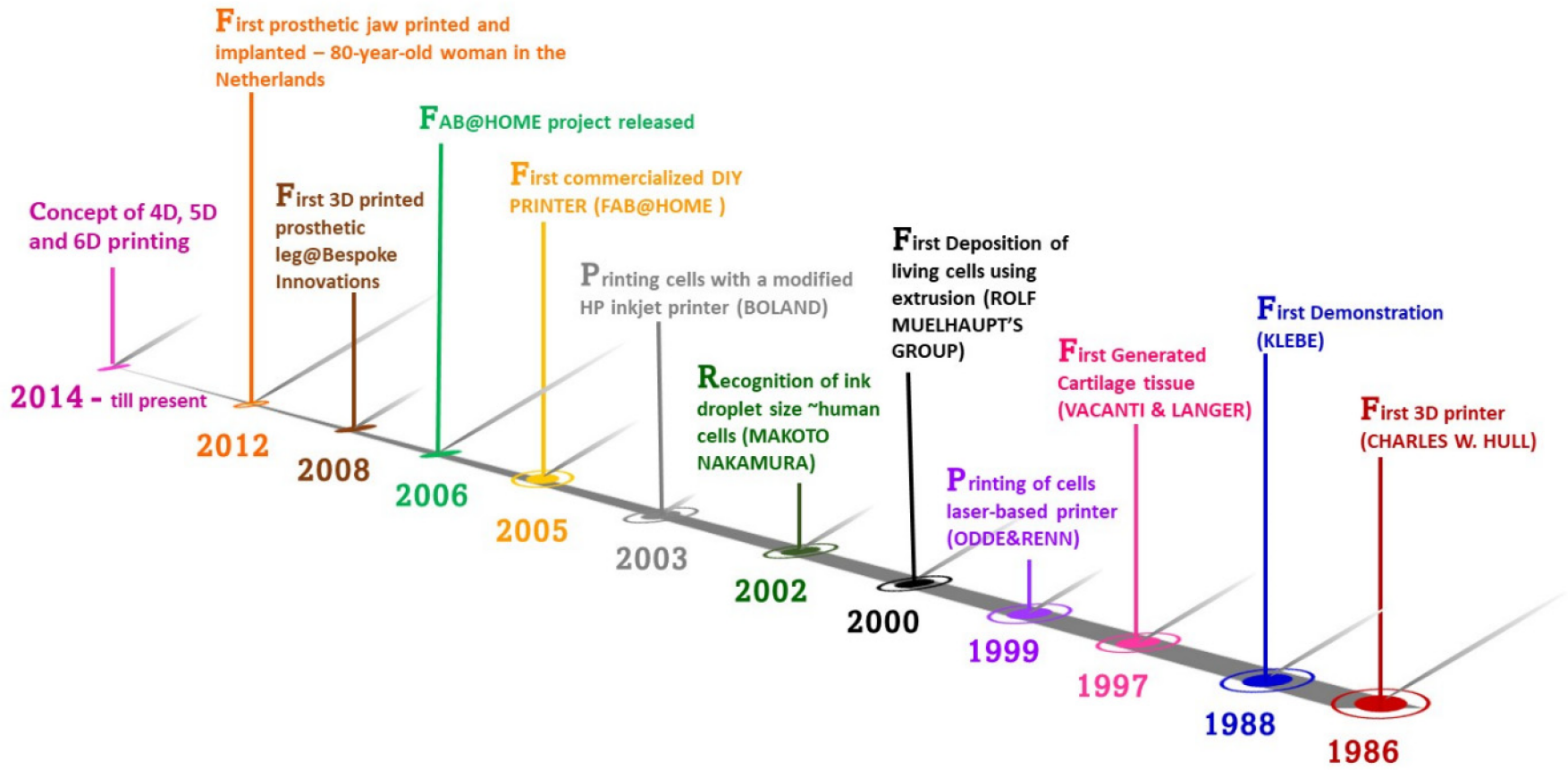
Schematic of milestones acquired in the 3D printing technology (2008, Bespoke Innovations, the first 3D-printed prosthetic leg; 2012, the first prosthetic jaw printed and implanted in an 80-year-old woman in The Netherlands).

Traditional AM techniques could not be effective in designing the 3D interconnected porous scaffolds with essential parameters such as porosity, pore size, and shape. Yet, given the materialization of 3D printing technologies and computer-aided design (CAD) techniques, it is effortless to control these parameters with high precision during the printing of the scaffold with biomimetic architectures. The advanced 3D printing method has vast potential in regenerative medicine for manufacturing patient-specific customizable porous scaffolds for bone tissue regeneration. It offers the possibility to engineer materials, bioactive molecules, and cells together to improve the potential of scaffolds to heal the defected bone. Figure 2 illustrates the milestones achieved in 3D printing technology, an attempt to manufacture 3D-printed porous scaffolds with or without cells, and commercial worldwide.

**Figure 3:**
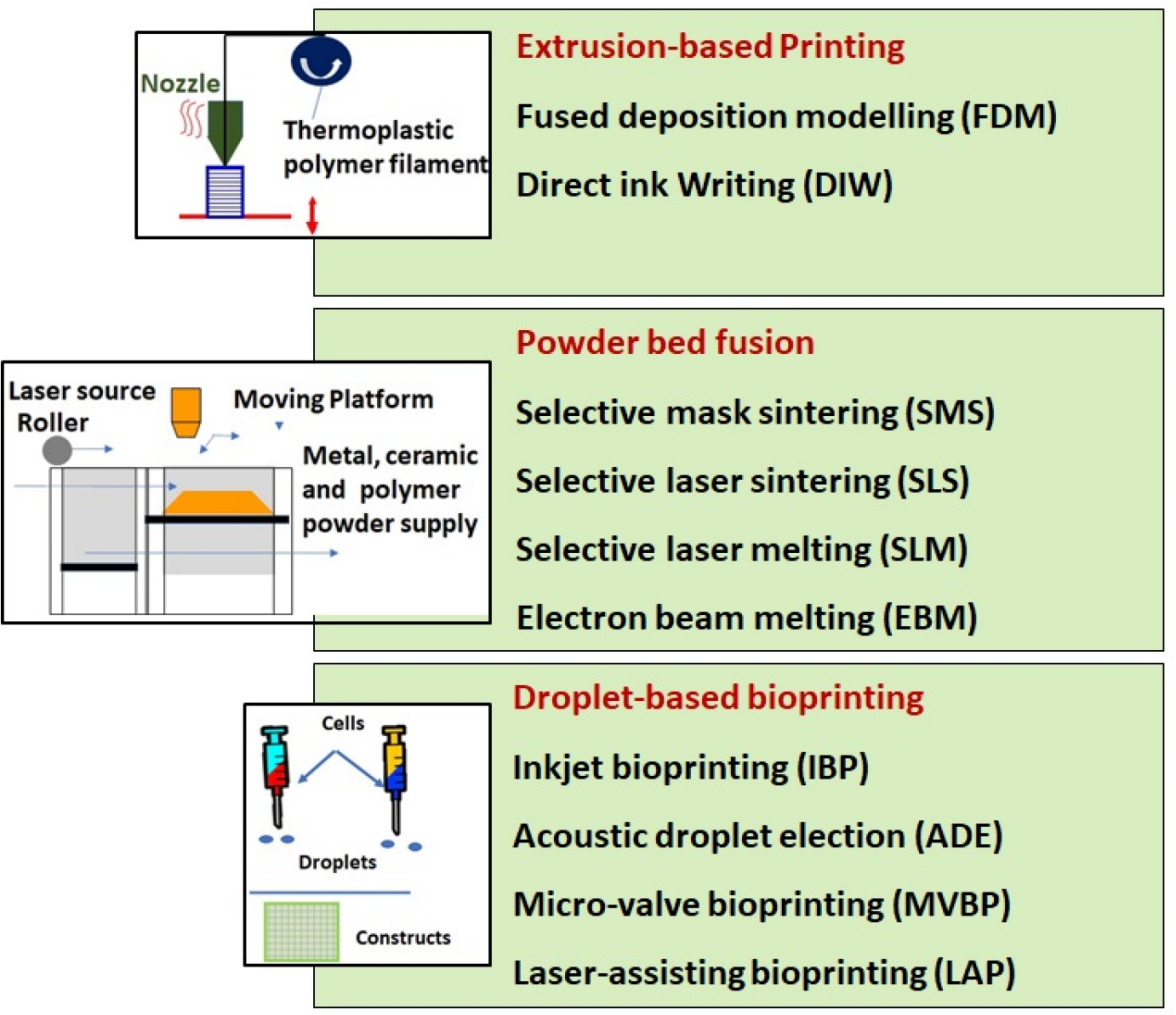
Schematics on the classification of 3D printing technologies extensively used for orthopedics. Three primary classes of 3D printing involve extrusion-based, which is further divided as FDM and DIW, followed by powder-based fusion, which is again classified down to SMS, SLS, SLM, and EBM, and last, the incorporation of cells in the printing which is mainly known as bioprinting based on the class of droplet-based categorized as IBP, ADE, MVBP, and LAP.

The classification of the frequently used 3D printing technologies for fabricating biodegradable porous scaffolds for orthopedic applications is presented in Figure 3. 3D printing technologies have gained significant recognition in orthopedics due to their continuous advancement to fabricate high-precision designs and control over the porosity, pore size, and intricate shapes. Various types of biocompatible materials are being explored to meet the existing challenges in bone tissue engineering. Hence, fabrication technologies are also continuously evolving as per the requirement of materials and their intended applications. In view of this, different 3D printing technologies have been demonstrated (Figure 3) to fulfill the demand for materials fabrication and enhance their capability to accommodate various functionalities for bone tissue engineering. Considering the working principle, 3D printing technologies can be categorized into three generalized techniques. These technologies are predominantly used for the fabrication of 3D antimicrobial scaffolds. Various metallic, ceramic, polymeric, and composite-based materials can be printed using these technologies. The following sections have elaborated brief details about these 3D printing techniques.

### 2.1 Extrusion-Based 3D Printing

**Figure 4:**
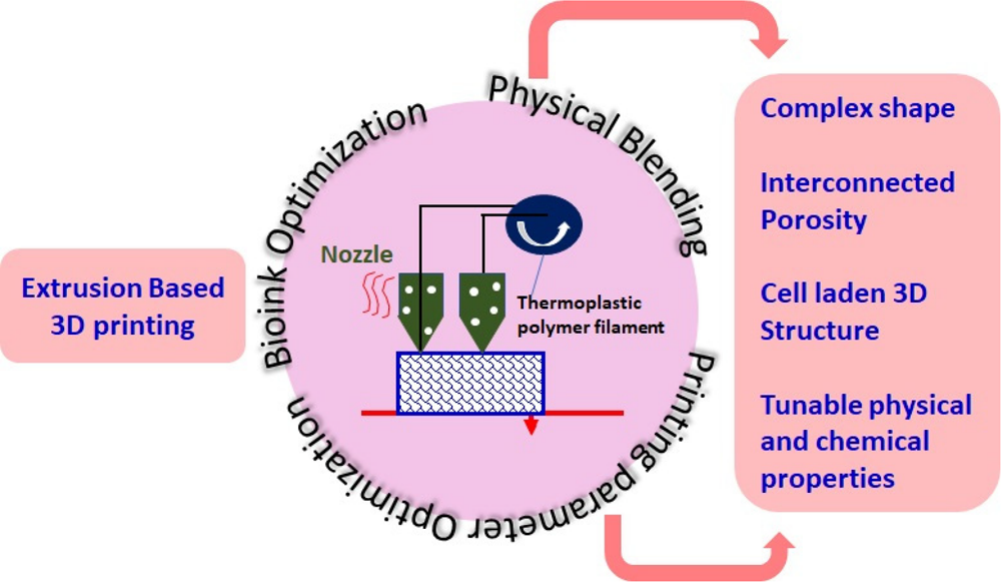
Essential parameters required for excellent printing of scaffolds via 3D extrusion-based printing of scaffolds with functional properties. Bioink optimization plays a crucial role in the material’s behavior regarding compatibility in the body environment. The physical properties of the designed construct depend on the structure, which is precisely controlled by the parameters. Uniform mixing of materials ensures the isotropic behavior of scaffolds.

Fused deposition modeling (FDM) and direct ink writing (DIW) are categories of 3D printing processes that involve extruding materials for structural support via a nozzle for fabricating layer-by-layer components.^18^ In the year 1989, Crump devised the first FDM^19^ to construct complicated geometries with excellent resolution. The process is primarily applicable to thermoplastic polymers such as polylactic acid (PLA) and poly-ε-caprolactone (PCL).^20^ The advantages of FDM involve no waste and pollution, which makes it an essential additive manufacturing method in orthopedic applications.^21,22^ Several other thermoplastic polymers, such as poly(l-lactic acid) (PLLA)^23^ and poly(vinyl alcohol) (PVA),^24^ are also used in FDM printing. Materials with various pore size ranges (80 to 190 μm and more than 300 μm) can be obtained through FDM.^25,26^ The pores are denoted by the fusion of multiple polymer layers, which results from interfilament spacing.^27,28^ The general design parameters essential for 3D extrusion-based printing are shown in Figure 4.

With the help of FDM, various polymeric/metallic composite-based 3D porous biodegradable scaffolds have been fabricated, for example, PLA/Fe,^29^ PLA/Cu,^30^ PLA/Cu alloy,^30^ PLA/Ag,^30^ PLA/Mg,^31,32^ PCL/Mg,^33,34^ etc. In this technique, the starting materials are polymeric beads, and powders of metal are mixed mechanically or by melting, followed by extrusion to produce filament. The fabricated filament is incorporated into a desktop printer to make a 3D porous structure with the required geometrical structure and various dimensions. Through FDM, complex interconnected pores and pore geometries can be developed. In one of the previous studies, PLA/Mg composite 3D scaffolds fabricated via the FDM technique showed a 3D interconnected porous structure that displayed outstanding printability and resolution with ∼66% porosity and a pore size of about ∼480 μm. Moreover, the excellent dispersion of Mg powders in the PLA matrix increased the surface roughness of the scaffolds,^32^ which enhanced cell adhesion properties.

The following are the pros (+) and cons (−) of FDM:

- (+) High surface finish
- (+) Manufacturing larger and more intricate shapes, scalable design is possible
- (+) High-speed printing
- (+) Simple manufacturing technique
- (+) Low investment cost
- (+) Low cost-to-size ratio
- (+) A wide range of materials can be printed
- (+) Able to produce multicolored parts using colored polymer
- (−) Overhanging parts are challenging to produce
- (−) Weak mechanical properties
- (−) The process is limited to only thermoplastics and layer-by-layer finish
- (−) Low resolution
- (−) The quality obtained is not good

### 2.2 Powder Bed Fusion (PBF)

The powder bed fusion technique is considered the gold standard for orthopedics.^35,36^ In this process, a metallic or ceramic powder layer is spread on the plate, followed by selective area melting using an electron beam or laser and then the sintering. Lowering the build plate allows the process to continue incrementally. A new layer of metal powder is applied over the previous layer, which is selectively melted or sintered. Further, the laser selectively deliquesces layer-by-layer, resulting in 3D sections. The pulverized material is unfolded over an antecedental joined layer, synthesizing it for subsequent layers process, resulting in indistinct than continuous output. The pulverized powder is delivered through a hopper, which is then spread evenly over the powder bed to provide space at the platform through a brush or roller. Selective laser melting (SLM), SLS, selective mask sintering, and electron beam melting (EBM) are widely used PBF processes.^37^ To fuse the powdered layers into one solid compact, the heat is used from the laser source (e.g., CO_2_ laser) in the SLS and SLM processes. In general, the SLM is operated at high temperatures compared to SLS, and EBM is carried out in a vacuum.^38^ Operating temperatures and the nonsuitability to low-temperature biodegradable metals are the major limitations of EBM technology.

Among the techniques described above, SLS is commonly used for fabricating porous 3D scaffolds with complex pore geometrical structures. It is a promising method to fabricate functionally gradient porous structures to imitate the geometry of bone tissue anatomically.^39^ Additionally, the scaffolds synthesized through SLS exhibits mechanical behavior closer to human trabecular bone.^40^ A 3D porous structure PLLA-polyglycolic acid (PGA)/GO-Ag was fabricated by SLS and showed an interconnected pore size of ∼500 μm.^41^ The reinforcement of graphene oxide (GO) enhanced the mechanical properties of the construct, while silver (Ag) particles contributed to improving the antibacterial efficacy. Interestingly, the addition of codispersing Ag/GO nanosystem into polymeric scaffolds showed a synergistic effect on the mechanical and antimicrobial properties of scaffolds. The compressive strength and elastic modulus of the codisperse GO/Ag nanosystem enhanced by 102% and 82%, respectively, compared to without one. These antibacterial and suitable mechanical properties of SLS-developed scaffolds might have potential for bone tissue engineering.

A satisfactory resolution, complex structure, and high-quality printing are powder bed fusion’s main advantages. This is one of the advanced methods for producing scaffolds for orthopedics. Use of a powder bed as support overcomes the difficulties faced by the supporting material. Despite advantages, the main drawback is a time-consuming technique that results in high costs and excessive porosity when the powder and binder are fused.

### 2.3 Bioprinting

**Figure 5:**
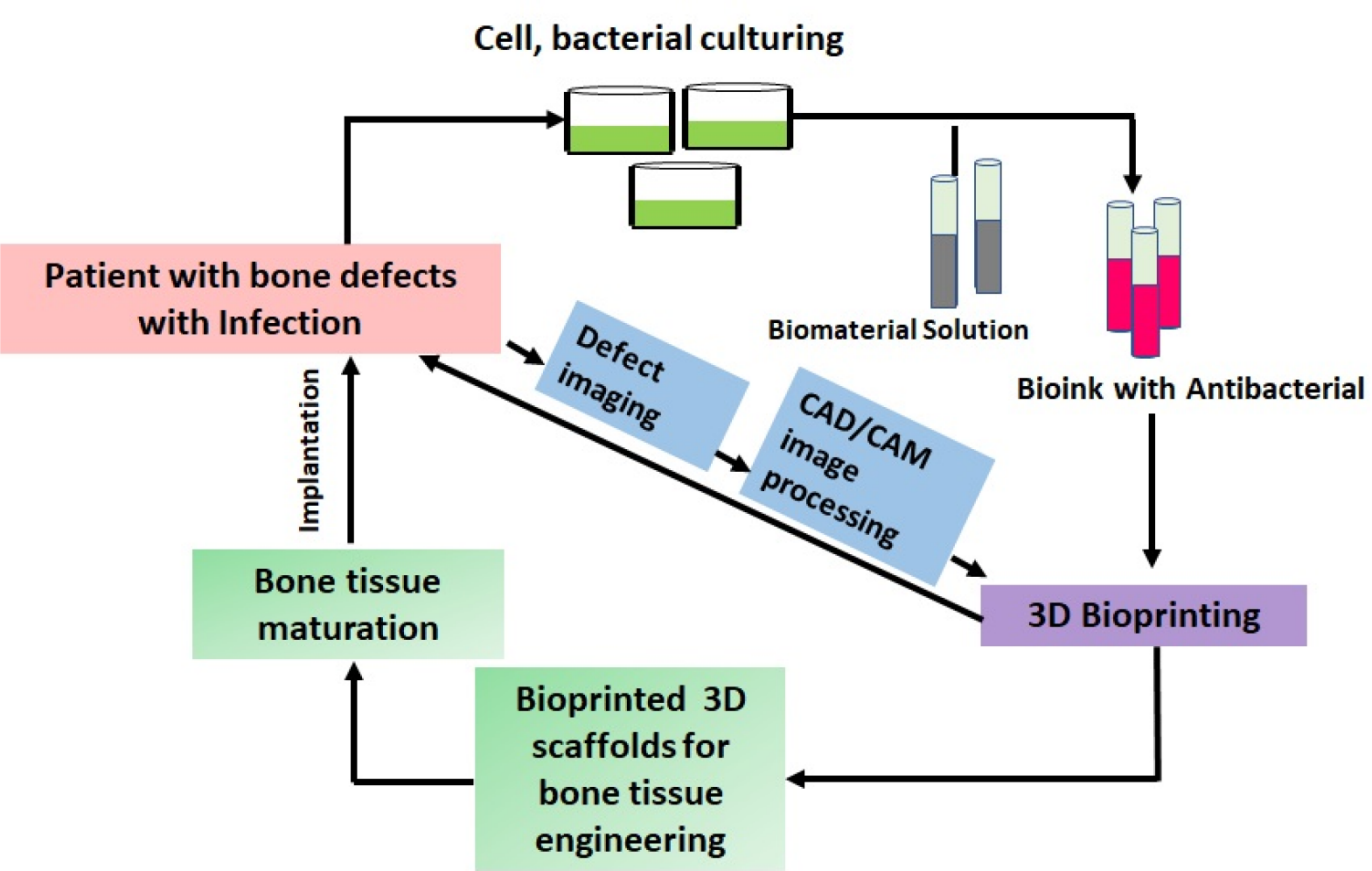
Schematic of the steps involved in fabricating antibacterial 3D scaffolds via 3D bioprinting for bone tissue engineering. The prebioprinting steps include preparing the design virtually through CAD software. Scanning and extracting the bone tissue image or capturing images from the bone organ can also be carried out for reconstructing the 3D models. The process involves slicing a 3D model sliced into 2D sections, rendering layer-by-layer.

3D bioprinting is a revolutionary and extended application of the AM method. 3D bioprinting has enormous potential for orthopedics and regenerative medicine. The definition of bioprinting is “the printing of a 3D structure by the deposition of biochemical, biomaterials, cells and bacteria onto a specimen in a defined pattern with the use of CAD and build-up processes”.^42^ The 3D construct of the antibacterial bone tissue scaffolds is printed via layer-by-layer deposition of biological materials, cells, and bacteria simultaneously. The three significant steps involved in 3D bioprinting are presented in Figure 5.

There are different categories of 3D bioprinting: (1) acoustic droplet election, (2) inkjet bioprinting, (3) laser-assisting bioprinting (LAP), and (4) microvalve bioprinting.

An antibiotic-loaded, biodegradable polymer scaffold can eradicate osteomyelitis and restore bone tissue. The scaffolds are promising candidates for the delivery of antibiotics in bone tissue engineering. In a study, 3D PLGA/HA nanocomposite scaffolds (porosity of 76.8 ± 3.7% and pore size of 307.6 ± 28.5 μm) were fabricated through fourth generation 3D bioplotter (EnvisionTEC GmbH, Germany) and grafted with quaternized chitosan (HACC) to impart the potential of antibacterial activity, repairing and restoring the infected bone defects.^43^ The composite scaffolds showed interconnected porous structures, which facilitate the space for cell penetration and growth of bone. In addition, the HACC-grafted PLGA/HA and PLGA/HACC nanocomposite scaffolds decreased the adhesion of bacteria and biofilm formation in both in vitro and in vivo experiments. Adenosine triphosphate (ATP) leakage assay study revealed that immobilizing HACC on the PLGA/HA scaffolds significantly disrupted microbial membranes.

Further, in a study, bioceramic (i.e., β-tricalcium phosphate) scaffolds (pore size 300–500 μm) were fabricated by 3D printing method (developed by the Fraunhofer Institute for Materials Research and Beam Technology) using a layer-by-layer approach with a triangle pore morphology for orthopedics.^44^ The bioceramic scaffold surface was modified with the homogeneous nanocomposite of antibacterial Ag, and GO was prepared via a liquid chemical reduction approach. The results revealed that the β-TCP scaffolds modified with Ag@GO nanocomposites showed outstanding antibacterial activity against Gram-negative bacteria. The highly porous structure formed an apatite layer composed of nanocrystals with a Ca/P ratio of 1.54, which helps with osteogenesis.

Similarly, the nano-MgO-modified polymer [poly(3-hydroxybutyrate-*co*-3-hydroxy valerate) (PHBV)] scaffolds with well-ordered and 3D interconnected microporous structures (pore size 300 and 800 μm) fabricated by selective laser sintering (SLS) showed excellent antimicrobial behavior, cell proliferation, and osteogenic differentiation.^45^ With the help of SLS, interconnected pores could be developed, and the addition of 5 wt % MgO could improve the compressive strength and elastic modulus by ∼96.18 and 52.34%, respectively, compared to the nonmodified polymer. Further, adding MgO caused oxidative and generation of reactive oxygen species (ROS) along with direct contact action causing mechanical damage to bacteria, which led to the enhanced antibacterial properties of MgO-modified PHBV scaffolds.

**Figure 6:**
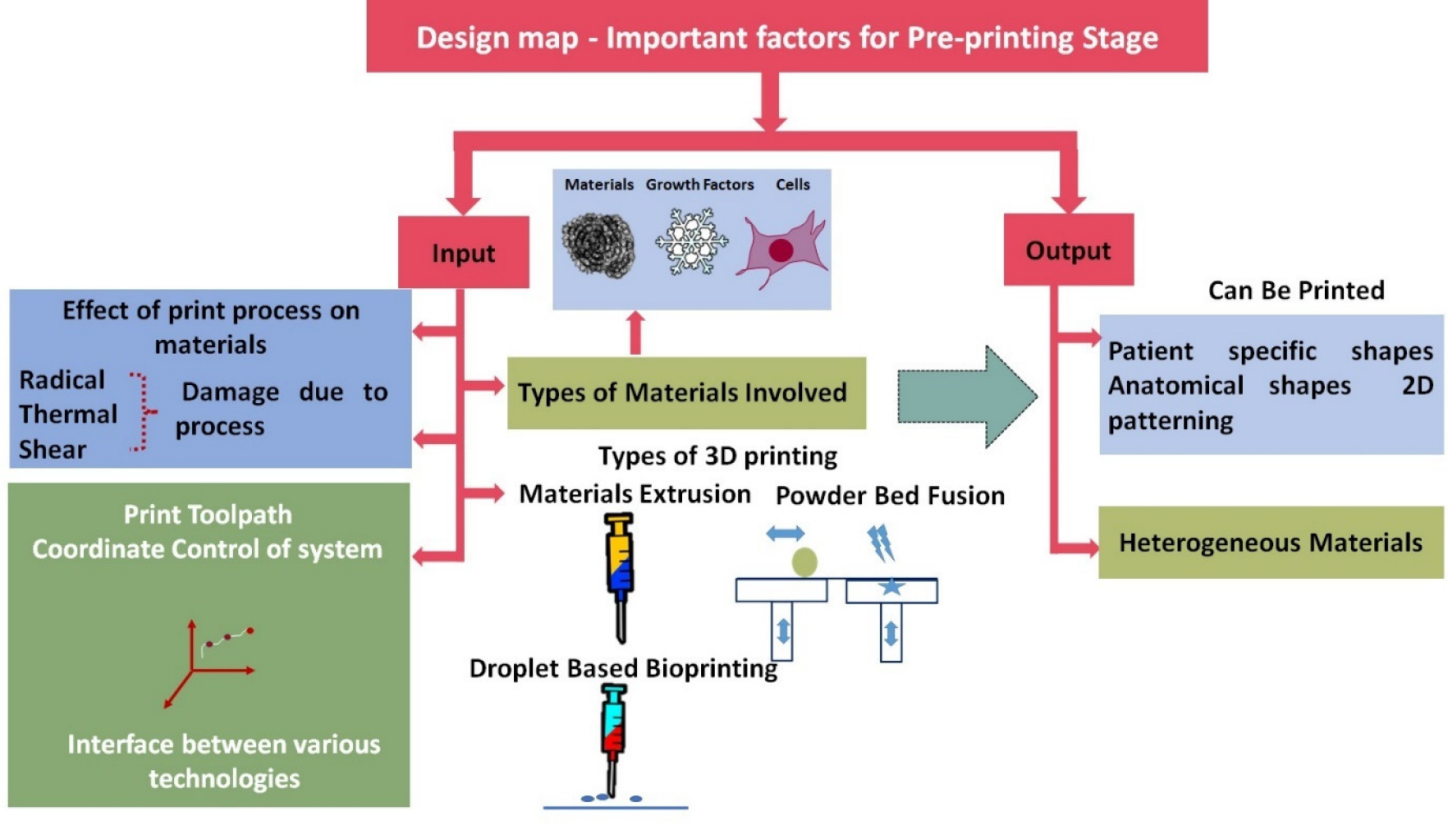
Schematic of a design map with important factors that need to be considered during the preprinting stage.

It was concluded from the studies that the type of bioink (i.e., biomaterials) and complex structure of the final construct play a noteworthy role in determining the properties of scaffolds in each bioprinting process. Thus, a design map needs to be developed while formulating the materials and types of bioprinting before the final printing of the construct. It might help in choosing the appropriate method and obtaining superior properties of the scaffolds based on the requirement. Figure 6 illustrates the design map required to consider during the prebioprinting of the scaffolds.

The selection of materials, the process of fabrication, and parameters to control the architecture of scaffolds are key points that need to be considered while designing scaffolds for orthopedic applications. It dictates the two aspects of the design map, i.e., input design factors and the corresponding outcome. In actuality, the selection of design factors from the input vertical may determine the outcome features of the designed scaffolds. In the case of bioprinting, the ink may contain biomolecules such as cells, growth factors, and natural polymers. Thus, selecting suitable printing parameters, including thermal, chemical, and mechanical factors, becomes paramount. In addition, the regulation of the size and shape of the printed architecture depends on the degree of accurate 3D design and their G-codes, which propel the specific movement of the printhead. The control over the print toolpath and coordinate may produce the proper shape with the preferred functionalities of the scaffolds. The patient-specific and anatomical shapes of the final products depend on the appropriate selection of input design parameters and the fabrication process.

#### 2.3.1 Essential Factors for Printing with Bioinks

**Figure 7:**
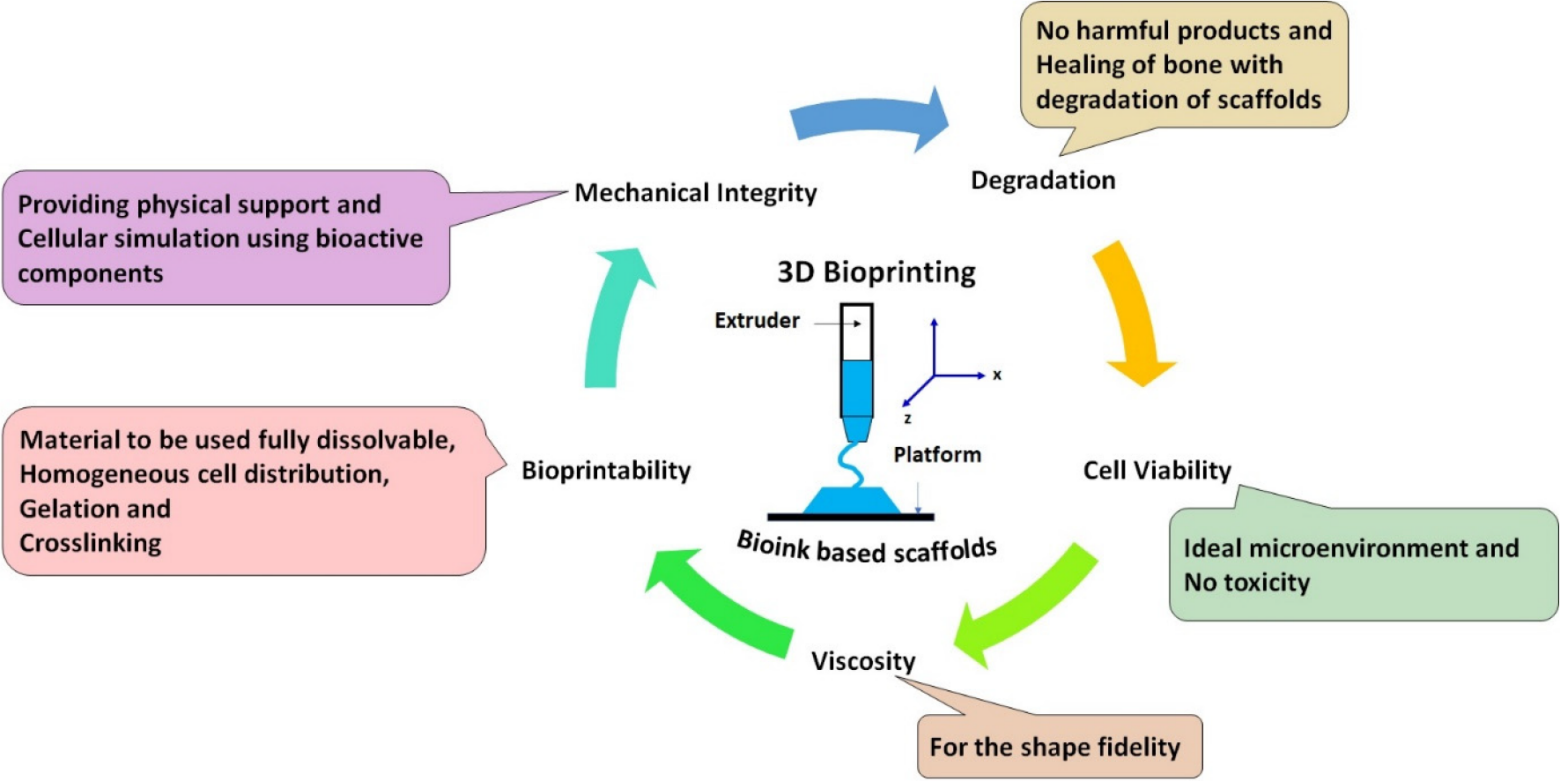
Schematic of the basic features and characteristics of an ideal bioink. The detailed criterion for designing the bone scaffold with bioinks in terms of viscosity properties, cross-linking, bioprintability, biodegradation behavior, cell proliferation, and mechanical integrity has been presented.

To build 3D-bioprinted structures that are functional, bioactive chemicals and living cells must be incorporated into the biomaterials. The discrete biomaterials class is bioinks which comprises cell materials, growth factors, signaling molecules as additives, and supportive scaffold materials, which biomimic ECM structure.^46^ Bioinks need to be stabilized rapidly to form the construct after layer-by-layer deposition, which will interact with cells and form tissue-like structures. The basic features and characteristics of the bioink required for the 3D printing of scaffolds are shown in Figure 7.

There are two types of bioinks, which are indicated as cellular bioink (with cells) and acellular bioink (without cells). Cellular bioink involves the encapsulation of cells to prevent damage during printing. Besides, it offers a suitable milieu for the maturation of printed orthopedic structures. It is very crucial to choose the appropriate bioink for bioprinting, as bioink offers an ideal biophysical and biochemical signal for proper cell–extracellular interaction. The following are the important characteristics required for the synthesis of bioinks:

- Temperature
- Gelation kinetics (cross-linking)
- Bioactive components and swelling, in addition to printability
- Affordability
- Practicality and resolution
- Mechanical/structural integrity
- Bioprinting/postbioprinting maturation times and biodegradability

Bioink can be synthesized from natural polymers, such as collagen, gelatin, chitosan, alginate, and fibrin, as well as bioceramics (HA, TCP, clays), which provide biocompatibility and have low immunogenicity along with high biodegradability. However, poor mechanical strength is the main limitation of natural polymers. Synthetic polymers, such as PCL, PLA, PLGA, PEG, and polyglycolide, can also be used in bioink printing to increase the mechanical properties. However, inadequate biocompatibility is the main limitation of synthetic polymers. Hence, researchers are blending materials using a mixture of natural and synthetic polymers that offer biocompatibility and mechanical stability.^47,48^

Bioinks can also be produced by incorporating various additives (such as graphene, silica nanoparticles, and nanoclays) into polymeric hydrogels, making a nanocomposite of bioinks with improved printability and mechanical strength. Literature dictates the mixed aminopropyl-modified silica nanoparticles with aldehydes-presenting oxidized alginate to foster the formation of reversible imine bonds^49^ which led to manifestation of shear thinning properties, enhanced printability and structural fidelity.

Recently, synthetic laponite nanoplatelets (BYK Additives Ltd.) showed interest in bone-mimicked composition (Mg_5.34_Li_0.66_Si_8_O_20_(OH)_4_]Na_0.66_) mixed with polymers such as kappa-carrageenan (kCA) and gelatin methacryloyl (GelMA), with good biodegradability, high specific surface area, and bone activity.^50^

### 2.4 Four-Dimensional (4D) Printing

3D bioprinting solely considers the initial state of the printed construct with an assumption of being static and inanimate. Natural bone tissue regeneration includes 3D constructs, microarchitectures, and an extracellular matrix (ECM), which possess unique functions that can only be achieved by dynamic changes in bone tissue. These functional dynamic conformational changes are ascribed to in-built mechanisms which respond to external or intrinsic stimuli that cannot be simulated via 3D bioprinting.^51^

**Figure 8:**
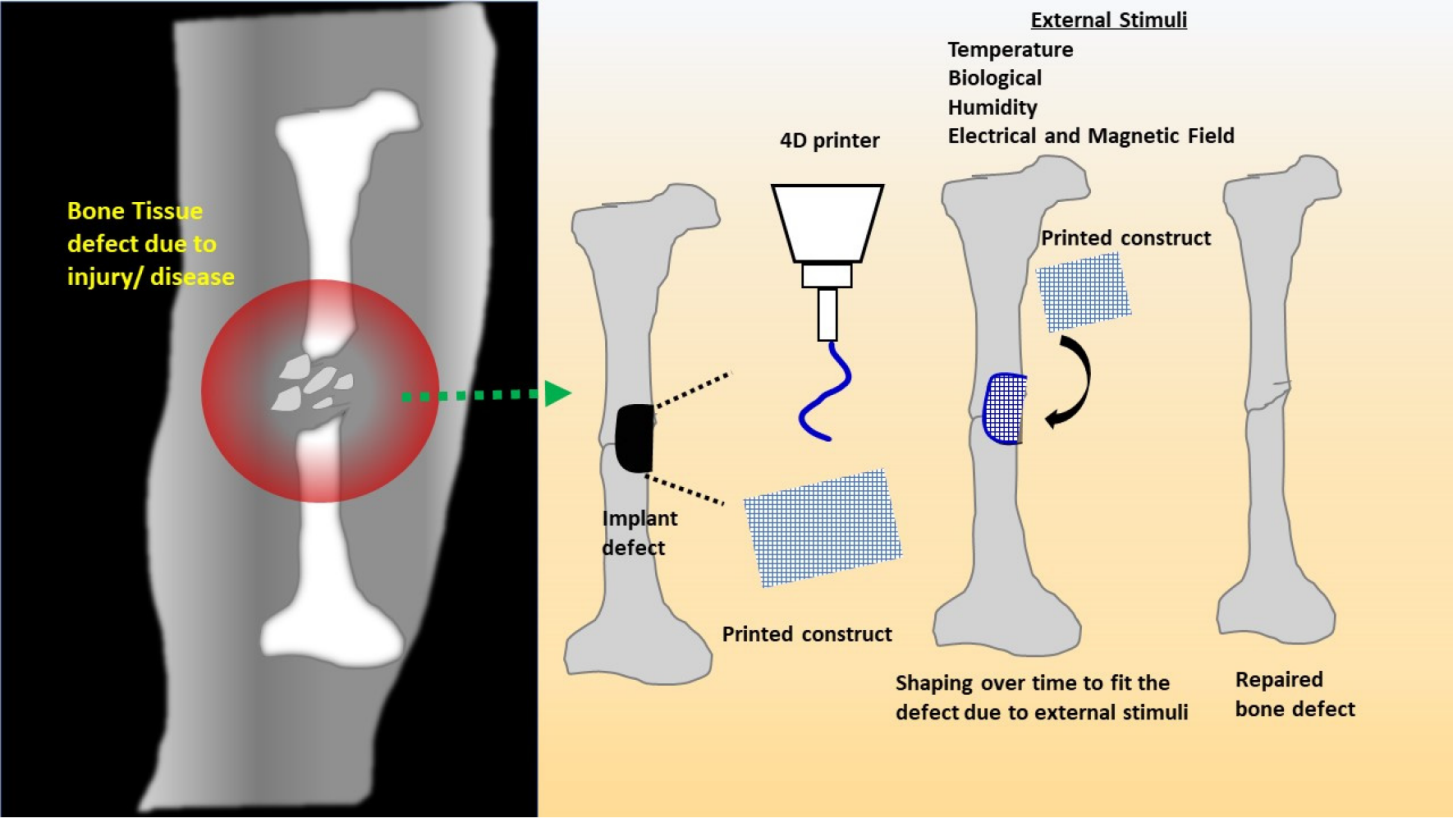
Schematic of 4D printing of a stimuli-responsive scaffold for bone defects. During the placement of a 4D-printed scaffold at the surgery site, it takes the shape of a defect or disease due to the effect of external stimuli present in the body’s fluid environment.

In 2014, the 4D printing technology was demonstrated at the Self-Assembly Lab at the Massachusetts Institute of Technology (MIT). It entails multimaterial prints that tend to transform over time and customized biomaterial systems that will change shape to another. Then, the technology was immediately applied to bone tissue engineering using the concept of time as the fourth dimension within 3D bioprinting, developing the new technology of 4D printing.^52,53^ With the help of 4D bioprinting, the biologically active scaffolds which are capable of dynamic functional conformational changes to stimuli with time, address the issues of 3D bioprinting (Figure 8).^51^

Generally, bone defects are of varying sizes and shapes, therefore 4D printing can be a helping hand in engineering the shape-fitting scaffolds to occupy the voids in bone defects.^54^ Considering the advantages of 4D printing technology for bone tissue engineering, an effort has been made in this section to focus on the existing literature on 4D printing technologies for fabricating 3D scaffolds to cure bone defects. For instance, PLA and HA porous scaffolds (pore size ∼700 μm and 30 vol % porosity) were fabricated via fused filament, in which the change in shape was stimulated after the application of heat.^55^ However, only mechanical and structural properties were studied in this study. The porosity was sufficient for cell infiltration and nutrients. The HA particles were nucleation sites during the ordering of molecular chains of PLA, forming a rigid phase, which decreases the mobility of molecule chains. Due to this, a temperature shift was observed from 53 to 57 °C for recovery stress. At 70 °C, a more rapid recovery stress development with a maximum recovery stress of 3 MPa was observed for PLA/HA composites. On the contrary, the shape memory effect for the composites is more than the human body temperature, i.e., 37 °C. It was observed with the addition of 15 wt % HA into PLA, a shape recovery of 98% was achieved. Thus, PLA/HA porous scaffolds, with their ability to shape recovery, are used as self-fitting scaffolds for repairing small bone defects. Such biomaterials are beneficial for repairing bone defects in which the construct changes the shape to fit in the void after implementation. However, the next challenge for the PLA/HA scaffold is to reduce the shape memory effect activation temperature without using any external heat source. Thus, using temperature as a stimulus is easy and highly sensitive for PLA-based polymers, but on the contrary, it is not suitable for body temperature and has a low biodegradation rate.

For infected bone defects, external specific biological signals and abnormal pathological factors can be used for designing the smart scaffolds.^56^ Another study was conducted using PLA, ferric oxide, and HA as materials and was fabricated through the 4D printing method followed by coating with collagen–dexamethasone (Col–Dex) layer for bone tissue regeneration.^57^ The porosity of the scaffolds lies between ∼53% and 62%. The scaffolds can recover into workable shapes and sizes as a result of the magnetic field. The process of shape recovery was covered within the 30s. It was observed that 4D-printed shape memory scaffolds with the HA as filler and coating of Col-Dex are efficient for defective bone repair. The advantages of using the magnetic field as a stimulus for the shape memory effect are remote control and not harmful to the cells, but eventually, they are complex control systems and very challenging to achieve sufficient magnetic gradient. However, no other studies were carried out using 4D printing for infected bone defects.

### 2.5 5D Printing

**Figure 9:**
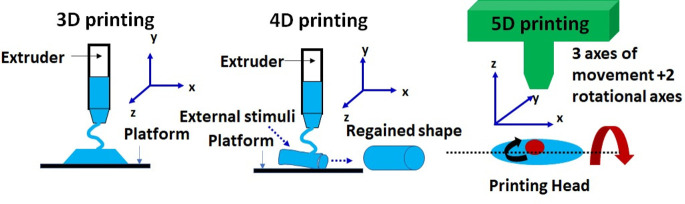
Schematic of the fundamental difference among 3D, 4D, and 5D printing technologies. The advent of 3D toward 4D and 5D printing will revolutionize biomaterials for bone tissue engineering. The schematic herein attempts to focus from 3-axis to 5D technology concepts giving stimuli responsiveness along with mechanical strength as a new function for 3D printers. The customized printer is a priory in designing the patient-specific scaffolds.

Complex shapes and strong scaffolds with curved surfaces are important in bone tissue engineering. The fundamental difference between 3D, 4D, and 5D printing technologies is schematically presented in Figure 9. In general, the 3D and 5D printing technologies produce a static structure; however, the 4D printing technology provides a dynamic structure that changes shape under external stimuli.

Of late, apart from 3D and 4D printing technology, another emerging potential additive manufacturing technique is “5D printing”.^58^ This technology consists of the print head and printable construct having 5 degrees of freedom; that is, it can produce curved layers instead of flat layers.^59^ The process involves the print head movement along with construction while printing. That is why the curve path of the construct can be obtained while printing rather than having a straight layer, as achieved in 3D printers. Hence, the main advantage is fabricating a curved layer with an efficient strength.

5D printing technique uses a five-axis printing method, unlike 3D printing, which uses only 3 axes for producing objects in multiple dimensions. The five-axis printing involves the movement of the print bed back and forth on two axes besides the X, Y, and Z axes of 3D printing methods. Therefore, 5D printing is promising for producing complex and stronger products compared to parts made through 3D printing. The 5D-printed model can produce curved scaffolds for bone tissue engineering because human bones have curved surfaces instead of flat ones. Thus, it has strong potential to fabricate complex artificial bones with adequate mechanical strength for the load-bearing application of these scaffolds.

The significant difference between 3D and 5D printing is the development of more substantial and curved part layers, whereas 3D printing creates flat surfaces; otherwise, both processes use the same input as the CAD file, 3D scanner, and 3D printing material. On the other hand, 4D printing is different from both technologies. It uses smart materials (which have thermomechanical properties) which can be programmable and modify their shape and function concerning temperature and time.^60^ To the best of our knowledge, until now, 5D printing has been applied only in lung tumor invasion to surrounding structures with an anatomic model.^61^ 5D printing technique was used by creating 3D models and data regarding tumor response and physiologic activity to induction therapy.

The deformed architecture caused by the tumor’s growth, infiltration of nearby structures, and alterations brought on by neoadjuvant treatment in complex thoracic surgical situations make surgical planning difficult. With the help of 5D printing, the effectiveness of treatment allows the visualization of the structures’ spare parts that were never involved clinically.

Height (*y*-axis), width (*x*-axis), depth (*z*-axis), change in tumor size (change in computed tomography [CT] measured dimensions), and physiology (change in positron emission tomography [PET]-measured 18F-fluorodeoxyglucose [FDG] avidity) were the five dimensions for printing that were included in 3D models along with the information about the tumor. Therefore, 5D printing would be one of the futuristic advanced AM techniques used to design patient-specific implants and scaffolds to address orthopedic infections.

## 3 Antimicrobial 3D-Printed Scaffolds

Autologous bone grafting is a commonly used practice for treating bone defects. Unfortunately, the strategy is correlated with the donor site’s morbidity, more surgical interventions, and a small portion of bone being pulled out from the patient. Much research has been carried out to substitute infected bone defects, which are functionally and structurally like actual bone. These 3D-printed biodegradable scaffolds will support the achievement of nutrient diffusion along with antibacterial behavior. Very few studies have been carried out to investigate the antibacterial behavior of recent promising biodegradable 3D scaffolds for orthopedics. By combination of the various strategies along with adjustment of the desired bone tissue properties, 3D-printed scaffolds could serve as a valuable tool in the field of orthopedics.

**Figure 10:**
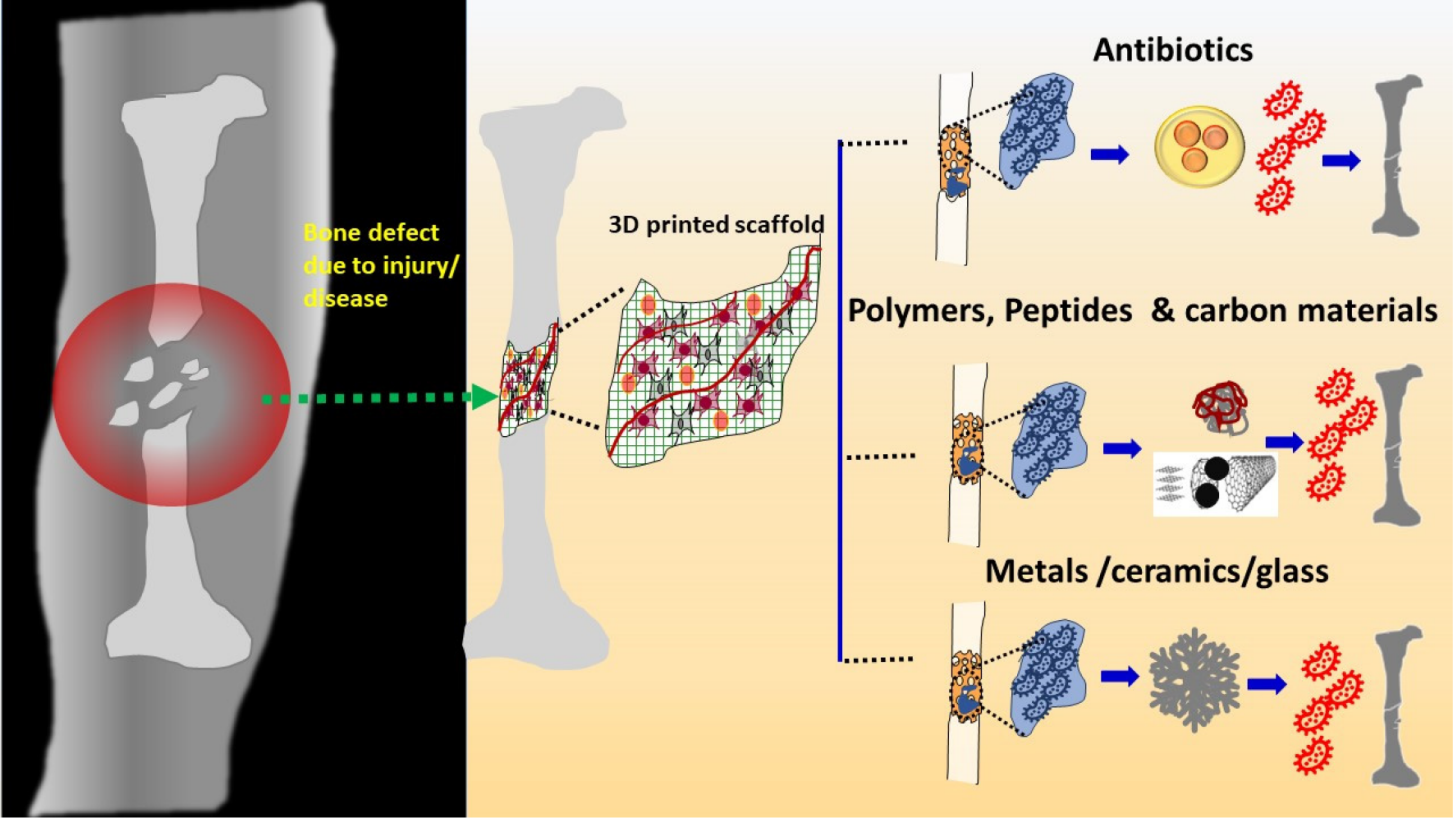
Design strategies of 3D-printed antibacterial scaffolds for orthopedic application (To accommodate the antibacterial properties of the scaffolds, various strategies, such as adding antibiotics in porous structure and using antimicrobial polymer, peptides, and carbonaceous materials to scaffold structure in the form of coating or impregnation into the porosity. In addition, several antimicrobial metallic and ceramic materials are also added to the structure to prevent bacterial infections).

3D printing can serve as a platform for designing customized scaffolds for treating infection. In addition, 3D bioprinting could be explored for treating specific infected bone defects with suitable mechanical, degradation, and biocompatible properties. Figure 10 presents strategies for designing biodegradable 3D-printed scaffolds with antimicrobial properties. The main consideration during fabrication is to control pore size and porosity, maintain good dispersion of antimicrobial agents, and, at the same time, minimize the overuse of antibiotics. Controlling the secondary particles, such as Ag nanoparticles and carbon-based nanomaterials, is required to maintain the biocompatibility of the 3D scaffolds. Hence, the intention of incorporating antibiotics, antibacterial polymer/peptides, carbon-based nanomaterials, metals, glass, and ceramics is to improve the antimicrobial properties of the 3D-printed scaffolds without sacrificing the biocompatibility while maintaining the optimum porosity to provide sufficient mechanical and degradation properties. The following sections deal with the efficacy of various strategies for designing biodegradable 3D-printed scaffolds with antibacterial behavior.

### 3.1 Incorporation of Antibiotics

Controlled drug release from scaffolds is a promising method for bone tissue engineering. This technique aims to focus on the issue and avoid the large concentrations of toxic antibacterial in the organisms or creating microbial resistance. Vancomycin (VAN) and Gentamicin (GEN) are glycopeptides active toward Gram-positive bacteria and are the commonly used antibiotics with the drug-releasing technique.

PCL scaffold coated with polydopamine (PDA) and adsorbed by polylactic acid-glycolic acid (PLGA) microspheres loaded with VAN was fabricated via 3D printing.^62^ The drug was initially released within 24 h, followed by sustained release from the microsphere (smooth, round, and uniform spherical shape) exceeding 28 days. The evaluations showed the inhibition of biofilm formation, maintaining the bacteriostatic effects against *S. aureus* for more than 4 weeks. Second, the adhesive properties of the 3D-printed biodegradable scaffolds with PDA coating scaffolds adsorbed the microsphere with better cell compatibility compared to those without coated PCL scaffolds.

Minocycline is another antibiotic used with bone cement and has shown excellent results in inhibiting infections.^63^ In another effort, 3D-printed PLA scaffolds were multifunctionalized with collagen (Col), HA, and minocycline hydrochloride (MH).^64^ The inhibition zone against *S. aureus* with a diameter of 26 mm was observed, whereas no inhibition zone was found without the antibiotic PLA-Col. The release of MH was effective against the bacteria, but also immobilization of the scaffolds confirms the antibacterial behavior. The combined 3D-printed scaffold with controlled antibiotics is the area that still needs to be explored, which could be advantageous while aiming at the antibiofilm formation. Similarly, in another study, 3D scaffolds loaded with minocycline drug and multiactive bioactive iron nanoparticles (IONPs) were used to promote and heal infected bone defects using 3D scaffolds fabricated via FDM.^65^ The minocycline profile showed that most of the drug was released within 24 h by the 3D HA-iron nanoparticle-based scaffolds. The antibiotics-loaded 3D scaffolds were able to inhibit *S. aureus* growth and any biofilm formation. The efficiency observed for 3D-IONPs (inhibition diameter) loaded drug is 83% in comparison to the control (inhibition diameter 29.3 ± 1.0). Some studies mention that using iron and HA nanoparticles boosts biofilm formation because of their nanostructures and topographical features. On the contrary, as shown by the mentioned study on 3D/HA/IONPs, they have better antibacterial effects. So, using iron nanoparticles to improve antibacterial properties is still debatable/controversial.

Other antibacterial drugs used in the literature are chlorhexidine, berberine, and rifampicin loaded into 3D-printed scaffolds for implanting bone-associated infections. In one of the studies, an alginate/calcium phosphate 3D-printed scaffold was loaded with berberine to fight against infection for bone tissue regeneration.^66^ The fabrication involves the combination of calcium phosphate, sodium alginate, and berberine to modulate the bioink and fabricate the 3D porous scaffolds (direct extrusion) and *in situ* cross-linking. As per the drug release profile, initially, the berberine was released rapidly from the scaffolds, followed by gradual release, and then stable. *In vitro* results showed that the berberine loaded possesses antibacterial capacity. Another drug is rifampicin (R), which is a first line antituberculosis drug but has the potential as an antibacterial drug, eradicating *S. aureus* (both stationary and adherent phase staphylococci). In a study, a rifampicin drug-releasing biodegradable polymer PCL scaffold using FDM printing was developed, and it showed the effectiveness of the drug elution against *S. aureus and E. coli*.^67^ The comparison of the drug release profile was made between rifampicin mixed with poly(methyl methacrylate) (PMMA) and mixed calcium phosphate cement (CPC). R combined with CPC showed rapid release up to 4–6 days, not dependent on the concentration, and completely released after 14 days, whereas in the case of PMMA, the release was at a particular concentration (2.5:1 ratio). A similar trend was followed in antibacterial studies. The R–PMMA group exhibited a bactericidal effect from 1 to 5 h but was lower at 7–24 h. On the contrary, the R-CPC group showed inhibition against bacteria up to 24 h, respectively.

With the help of 3D bioprinting, cell-laden and doxycycline (DOX)-loaded PCL and mesoporous bioactive glass (MBG) scaffold was fabricated with a double nozzle.^68^ DOX was loaded into the channels of MBG, which was further mixed with molten PCL, allowing the sustained release of the antibiotics from the scaffolds. The drug release profile of DOX shows the burst release by day 1–150 μg, further released cumulatively reaching up to ∼400 μg after 7 days and ∼600 μg after 21 days, respectively. The spread plate analysis showed a decrement in *E. coli* bacteria adhering to the scaffold surface.

Eventually, various studies have been proposed for antibacterial activity by loading synthetic antibiotics into 3D-printed scaffolds that impede bacterial adhesion and biofilm formation, improving the osteogenic property. Although antibiotics are the most effective way to deal with the problem of bacterial adhesion so far, the multidrug resistance of microbes is still a challenging task. The controlled release of antibiotics with long-term antimicrobial action can be achieved by adequately designing and manufacturing 3D-printed scaffolds. Additionally, antimicrobial features of scaffolds might be improved by engineering stimuli-responsive materials that could react to infections, release minute antibiotics directly to an affected area, and destroy the bacteria.

### 3.2 Incorporation of Antimicrobial Polymers and Carbonaceous Materials

Chitosan, cellulose, chitin, and various polysaccharides are well-known for their inherent antimicrobial properties. 3D-printed porous scaffolds based on PLA/gelatin/nano-HA and a peptide (ponericin) were investigated for antibacterial effect by mixing with 3 mL of *S. aureus* and *E. coli* in 4.0 × 10^4^ bacteria/mL LB culture medium for 18 and 24 h. It was observed that, at 250 μg/mL, the best antibacterial activity was attained for *E. coli*, whereas for *S. aureus*, it was 500 μg/mL at 18 h. Hence, it was concluded that adding ponericin into the scaffold was more sensitive toward *E. coli* compared to *S. aureus*.^69^ The hydrophilic nature due to the modification of polydopamine and affinity toward serum improved the antibacterial effect.

In another study, a 3D aerogel-based hybrid scaffold was fabricated via a micro extrusion-based 3D printing method followed by freeze casting using antimicrobial peptide-modified silk fibroin (SF) and silica. This composite scaffold showed antibacterial activities against Gram-positive and Gram-negative bacteria (minimal inhibitory concentration (MIC) values of 30–60 μg mL^–1^ for *E. coli*.,).^70^ The scaffold possesses a positive charge, which is electrostatically interacting with the negative charge membrane of bacteria, impairs the integrity of the membrane, and causes leakage of cytoplasm content, which leads to the death of bacteria. 3D printing technology was also used for hydroxypropyl trimethylammonium chloride chitosan (HACC) incorporated PLGA/HA scaffolds. The *in vivo* study was performed on the rabbits with a femoral condyle defect model injected with *S. aureus.* A considerable improvement was observed in the antimicrobial activity (against S. aureus) and regeneration of bone for the infected bone defects [63]. The authors have shown an antibacterial efficiency of∼ 26% for HACC scaffolds. The HACC-grafted composites decreased the bacteria numbers at 4 h and inhibited formation at 24 and 48 h compared with HACC-free scaffolds. Interestingly, to monitor the bacterial burden of scaffolds, the bioluminescent *S. aureus* strain (Xen29) was fabricated and immunized over the scaffolds after *in vivo* implementation. The observation over time of the *in vivo* bioluminescent signals showed the antibacterial activity ability of scaffolds until 14 days. The study lacked the investigation of antimicrobial behavior against Gram-negative bacteria (*E. coli*) for broadening the application of scaffolds for clinical application.

In another study, BMP2 mimetic peptide (BMP2-MP) and antimicrobial peptide PSI10 (RRWPWWPWRR) were incorporated into HA binding domain (HABD) to boost up the antibacterial and osteoinduction adeptness of HA scaffolds.^71^ The inhibition zones of bacteria of PSI10@HA and PSI10/HABP@HA scaffolds on *E. coli* were 7.97 ± 0.58 and 14.47 ± 0.89 mm, respectively. A similar trend was observed in the *S. aureus* group; the inhibition zone of bacteria of the HABP/PSI10@HA scaffold was 1.85-times that of PSI10. This enhancement was ascribed to the binding potential of HABP and HA materials, hence leading to the strong binding of PSI10/HABP antibacterial peptides with the HA particles’ surface.

Carbonaceous materials are also known for their physical and biological properties, for example, antibacterial activity and osteogenic behavior (able to express many genes).^72^ A small composition of carbon nanomaterials can improve the physical, mechanical, wettability, electrical, thermal, water diffusion, cell adhesion, proliferation, antibacterial, and biocorrosion properties of polymers.^73−76^ Commonly, scaffolds are fabricated using carbon nanomaterials as reinforcements with improved antibacterial activity. Recently, 3D-printed electroactive scaffolds made of PCL with reduced graphene oxide (TrGO) were explored for bone tissue engineering.^77^ It was observed that implementing an electrical stimulus of 30 V for 3 h to the rGO scaffolds entirely eradicated the *S aureus* growth in comparison to pure PCL scaffolds without rGO, which possesses bacterial attachment even after electrostimulation. Antibacterial results showed that the PCL/TrGO scaffolds could reduce 26% of bacteria in comparison to pure PCL due to the hydrophilic nature of TrGO composites. *S. aureus* has proteins and hydrophobic characteristics in its cell walls, such as carboxylic phosphatase and teichoic acids,^74^ so adhesion is hydrophobic. Another reason is surface topography; the sharp and narrow peaks achieved on the surface of PCL/TrGO scaffolds also bestowed the bacteria’s detachment of bacteria without electrical stimulation. 3D-printed scaffolds with gelatin, chitosan, rGO, and tricalcium phosphates also displayed antibacterial behavior against *S. aureus* and *E. coli* without affecting the osteoblast activity.

On the other hand, GO also exhibits remarkable antimicrobial behavior, although it has low electrical conductivity. GO was used as reinforcement with various concentrations in PCL with a 3D-printed fibrous scaffold prepared by a layer-by-layer assembly technique.^78^ The scaffolds improved the antimicrobial properties (against Gram-positive and Gram-negative bacteria) and simultaneously promoted cell adhesion. The highest concentration of GO in the scaffolds resulted in the death rates of *S. epidermidis* and *E. coli* up to ∼80% after 24 h of contact. The graphene-based materials are considered for antibacterial features, owing to the physical and chemical interactions of graphene sheets with microbes. The effect is affiliated with induced oxidative stress or disruption of bacteria physically; mechanisms are activated after 2 h and exacerbated 24 h after contact. Two significant effects of GO could have ensued as the bacteria were exposed to basal planes, such as (i) electron transfer between graphene and the bacterial membrane and (ii) oxygen adsorption on the graphene edges and defective sites. The simultaneous action of electron transfer and oxidative stress leads to the death of bacteria. Aside from the above events, the physical insertion of graphene-based material’s sharp edges passes through bacterial membranes creating a nanoknife effect followed by the breaking up of bonds between proteins, and pore formation might also occur. However, oxidative stress and electronic destabilization were proposed as the primary mechanisms responsible for the antimicrobial activity of GO. There are limited studies on the immobilization of GO in PCL compared to that of GO dispersions. The interaction of GO with various polymers might induce graphene platelets to be exposed differently, which might exert different antibacterial behavior. The addition of graphene with various oxidation degrees, lateral size, and thickness should also be explored along with multiple patterns of scaffolds via 3D and 4D printing. Moreover, it will be an interesting challenge to expose the graphene-based scaffolds to different bacteria that are involved in implanted scaffolds, such as *S. aureus or P. aeruginosa*, or in a mixed inoculum.

Indeed, adequate measures were proposed to deal with the bacterial attacks, with substantial scientific advances achieved in polymeric antibacterial materials. Despite that, some challenging issues still remaining. The leaching of antibacterial polymeric materials is detrimental to the human body and the environment. Specific polymeric antibacterial biomaterials even kill common bacteria and cells.^79^ These substances have ill-defined diseases and congenital malformations due to their prolonged use. The leaching of a subinhibitory concentration of antibacterial agents will facilitate bacterial growth. On the utilization of antibacterial carbonaceous materials has increased dramatically; it is too soon to use carbonaceous materials to compete with commercially available antimicrobial peptides in practical applications. The reasons are the presence of heterogeneity and impurities, which could affect the antibacterial performance to some degree. Additionally, there has not been enough research carried out on the potential toxicity of carbonaceous materials to human cells and tissues. Future research will therefore concentrate on developing carbonaceous materials that can be produced in large quantities and have high purity, homogeneity, and acceptable toxicity against bacteria while minimizing their toxicity to human cells.

### 3.3 Incorporation of Metals/Ceramics/Glass Nanoparticles

The addition of a small composition of metal nanoparticles to the base materials drastically affects the antimicrobial properties of the scaffolds. Embedding these antimicrobial nanoparticles in scaffold materials elevates the capability to treat infected bones. Along with the antibacterial properties, the degradation behavior of these nanoparticle-embedded scaffolds is also tailored, which is also an essential property required to augment biodegradable, antimicrobial scaffolds for bone tissue engineering.

Ag, gold (Au), Zn, strontium (Sr), or Mg nanoparticles have been investigated for their antimicrobial properties in scaffolds.

The scaffolds with titanium dioxide (TiO_2_) nanotubes and Ag ions are incorporated on the surface. The antibacterial effect was observed against *S. aureus* for 2 weeks.^80^ The scaffolds with a higher concentration of Ag (0.5M) showed an antibacterial efficiency of ∼100% on day 1. The samples with lower concentrations of Ag (0.02M) and without Ag showed an efficiency of ∼80%. The study used a higher concentration of Ag (0.5M), which added a certain level of cytotoxicity after day 1. In a recent study, the Ag ions were encapsulated into an FDM-produced PCL matrix.^25^ The PCL scaffolds with Ag showed an inhibition zone of 20.4 ± 1.7 mm, presumably due to ROS and silver diffusion from the scaffold to soft agar. Bactericidal behavior was also assessed via a liquid test in which scaffolds were soaked in a bacterial solution of 10 mL. A reduction of bacteria by ∼90% was observed. Further, the adsorption results in the silver released in the bacterial suspension are very small (<0.1 mg/L).

Because the lower concentration of Ag nanoparticles on the scaffold surface needs a longer contact time to analyze the bactericidal activity, such as 24 h in comparison to 6 h. Based on these findings, it appears that the concentration of Ag nanoparticles (Nps), the antibacterial agent, determines the level of antibacterial activity. Further, a bactericidal study was performed to analyze the capability of the scaffolds to disinfect the bacteria grown on the implanted scaffolds.

Zn is also known as an antibacterial agent, along with osteogenesis and vascularization. The release kinetic profile of Zn ions in a controlled manner is of prime importance for the use of bone tissue scaffolds. For instance, the PCL/SiO_2_–CaO–P_2_O_5_–ZnO (mesoporous bioactive glass) MBG scaffold was fabricated by 3D printing. The MBG, with its mesoporous structure, induced better degradation, bioactivity, and antibacterial characteristics.^81^ The Zn substituted MBG scaffolds showed a drop in the percentage of bacteria (∼30%), which offers a remarkable loss in bacteria, clearly indicating that Zn hinders the formation of biofilm by *S. aureus* and ascendancy increases with a percentage increase of Zn in MBG scaffolds. Zn hinders the activities in the bacterial cell, for instance, glycolysis, acid tolerance, and transmembrane proton translocation. Additionally, Zn has shown better antibacterial effects even at lower concentrations in comparison to other antimicrobial agents. Several other studies incorporated Zn ions in the scaffolds to enhance biocompatibility. However, none of them explored antibacterial properties.^82^

Another study was conducted using Au nanoparticles incorporated in calcium-deficient hydroxyapatite (CDHA), which manifested efficaciously antibacterial properties against *Micrococcus luteus* for bone tissue engineering.^83^ The antibacterial effect was observed by using the dilution technique. Au incorporated scaffold was able to inhibit the bacteria by 60%. Even the lower composition of Au was able to hinder bacteria. The author compared the antimicrobial activity of Au with the available antibiotics, for instance, gentamycin, ceftriaxone, streptomycin, tetracycline, and ampicillin, and observed that the Au scaffold was 2 and 3 orders better than these antibiotics. Further, the antimicrobial behavior was also carried out at the site-specific implant, and inoculation of agar test found no inhibition zones, which indicates Au nanoparticles were not released. Hence, the authors confirmed that the Au was effective at the implant surface, which is a beneficial feature as the antibacterial behavior is located at the site of infections and overcomes drug resistance bacterial drawback. Several studies have been carried out on 3D-printable and biodegradable Mg-based scaffolds for bone, but unfortunately, none considered the antibacterial behavior of Mg-based scaffolds for bone tissue engineering.

### 3.4 Near-Infrared (NIR) Light Photothermal Therapy (PTT)

Photothermal therapy (PTT) uses photoheat to create topical hyperthermia to kill aberrant cells.^84^ It has the advantages of being minimally invasive, having excellent spatial-temporal precision and having little to no damage to healthy tissue.

NIR light has a larger penetration depth than UV or visible light, making it the optimum frequency for PTT. This is because NIR light has higher photon energies, less tissue absorption and scattering, and deeper penetration. Although various tumor types, such as prostate cancer, malignant liver tumors, colorectal carcinomas, and gliomas, can be treated with PTT using only a laser, the high operating power and complex laser devices may raise safety and practicality concerns. To eliminate the resistance provided by traditional antimicrobial drugs, PTT can kill both drug-sensitive and drug-resistant cells through thermal ablation or damage to the reactive species. NIR-II is also preferred over NIR-I in promoting severe infections in deep tissues.

A study reported the photothermal activity of 3D-printed forsterite scaffolds (made of polymers and HA) against bacterial infections. The polymer-derived forsterite scaffolds were found to possess the photothermal *in- vitro* antibacterial capability under 808 nm laser irradiation to inhibit *S. aureus* and *E. coli* growth. The antibacterial efficiencies of the scaffolds against *S. aureus* and *E. coli* were enhanced by 96.1% and 95.6%, respectively.^85^

A customized photothermal MXene-based hydrogel scaffold (GelMA/-TCP/sodium alginate (Sr^2+^)/MXene (Ti_3_C_2_) (GTAM) hydrogel) was developed by using 3D printing. *In-vitro* analysis showed that the GTAM scaffolds were able to inhibit the growth of Gram-positive and -negative bacteria via NIR light. Besides, 3D bioprinting was performed with the rat bone marrow mesenchymal stem cells assorted with GTAM bioinks. Cell-laden 3D-printed GTAM scaffolds showed accelerated bone formation ability. The temperature of GTAM0.3 could reach 56 °C under wet conditions with NIR irradiation (808 nm, 1.5 W cm^–2^). MXene-based scaffolds showed antibacterial properties by depleting the *S. aureus and E. coli* membranes through NIR irradiation.^86^

The key findings and summary of state-of-the-art 3D-printed scaffolds with respect to the fabrication method, antibacterial properties, antibacterial mechanism, and biocompatibility are presented in Table 1.

**Table 1:** Key Findings on Antimicrobial Behavior of State-of-the-Art 3D-Printed Scaffolds

Material	3D Printing Technique	Efficiency and Antibacterial Mechanism	Biocompatibility	Ref
Scaffolds with Antibiotics				
PCL composite scaffolds with vancomycin-loaded PLGA	Biological fused deposition 3D printer (Shanghai Fuqifan Electromechanical Technology Co., Ltd.)	99% *Staphylococcus epidermidis*	Rabbit bone MSCs animal model	(62)
		Cell wall inhibition	The scaffolds were conducive to cell attachment and proliferation	
Macroporous agarose/Nanocrystalline hydroxycarbonateapatite (nHCA) scaffolds the drug Vascular Endothelial Growth Factor (VEGF) and cephalexin	GELPOR 3D method	Value not mentioned for *S. aureus*	120% cell viability MC3T3-E1 preosteoblast	(87)
		Prevents the synthesis of nucleus acid	The hydrophilic nature was suitable for cell adhesion and proliferation	
			No *in vivo* study conducted	
PLA/collagen/minocycline/HA	FDM	100% *S. aureus*	40,0000 AU fluorescence hMSCs	(64)
		No inhibition zone for PCL	No *in vivo* study was conducted	
		It inhibits protein synthesis, cytoprotective effects		
PGLA/Polyetheretherketone acid scaffolds	Selective laser sintering	55.71% *E. coli* and 15.84% *S. aureus*	Cell viability is not mentioned	(88)
		It disturbs the synthesis of peptidoglycan and hinders protein synthesis	Dapi staining hFOB1.19 osteoblast cells	
			No *in vivo* study was conducted	
Scaffolds with Antibacterial Polymers/Peptides				
HACC-grafted PLGA/HA scaffolds	fourth generation 3D Bioplotter (EnvisionTEC GmbH, Germany)	Not mentioned *S. aureus*	>150% cell viability. No toxic nature was found in Rat and Rabbit animal model	(89)
PCL/PDA/AgNPs scaffold	FDM	97% *S. aureus* [Ag(NH_3_)_2_]^+^ ROS	>150% cell viability. No toxic nature found Rabbit BMSCs/Rabbit animal model	(90)
		Breakage of cell membrane of bacteria		
PLA/gelatin/HA with ponericin	FDM	100% *E. coli* and 100% *S. aureus*	110% cell viability	(69)
		The antibacterial mechanism is not mentioned	No toxicity was obtained during *in vitro* MC3T3-E1 preosteoblast	
			No *in vivo* conducted	
EPL/PCL/HA scaffolds	FDM	92.42% *S. aureus* and *E. coli*	150% cell viability. No toxicity was obtained during *in vitro* MC3T3-E1 preosteoblast	(91)
		Electrostatic adsorption of EPLbreaks the cell membrane of bacteria	No *in vivo* conducted	
Silk fibroin and silica NP-modified peptide	Microextrusion followed by directional freeze-casting/drying techniques	98–99% *S. aureus* and *E. coli*	150% cell viability. No toxicity was obtained during *in vitro* MC3T3-E1 preosteoblast	(70)
		Induces pH increase and osmotic pressure	No *in vivo* conducted	
PCL/rGO	3D printer (Envionstec, fourth generation, Germany) and high-temperature accessories	89% *E. coli* and *S. aureus*	110% cell viability. Nontoxic Human BMSCs	(77)
		Electrons flow through the electroactive conductive biomaterial, causing the electrophoretic forces to have an antiadhesive effect, increasing the mobility of bacteria, and leading to desorption on 3D-printed conductive scaffolds	No *in vivo* conducted	
Chitosan/rGO/gelatin/TCP	Extrusion-based 3D printer	80% *E. coli* and *S. aureus*	90% cell viability. nontoxic hOB human osteoblast	(92)
		By interacting its positively charged amine groups with the electronegative residues on the surface of the bacterial cell wall, increasing the cell wall’s permeability and allowing intracellular constituents to leak out, dissipating ionic gradients within the microorganisms	No *in vivo* conducted	
Scaffolds Incorporated Metals/Ceramics/Glass				
Ag- zincosilicate zeolite scaffolds	Extrusion-based 3D printing	99% *E. coli* and *S. aureus*	60% cell viability. No toxic nature MC3T3-E1 preosteoblast	(93)
		ROS Induction breaks the cell membrane cell membranes	No *in vivo* conducted	
PLGA/Cu	Fourth generation 3D Biplotter (EnvisionTEC GmbH, Germany)	Not mentioned *S. aureus*	Murine MSCs/Rat nontoxic nature in animal model	(94)
		It disrupts the cell wall of bacteria, inhibits the DNA; ROS induction		
PLA/halloysite nanotubes (HNTs) loaded with Zn	FDM (ENDER 3 printer)	Not mentioned *S. aureus*	MC3T3-E1 preosteoblast	(95)
		It destabilizes the membrane and enhances permeability		
NPs		It deactivates the enzymes and nucleic acids		
		ROS induction		
CaPs	Direct extrusion	99% *S. aureus*	80% cell viability L929 fibroblast cellsnon nontoxic nature in animal modeling animal model	(66)
		Reducing the number of bacteria fimbriae		
Forsterite scaffolds	Fourth 3D Bioplotter (EnvisionTEC GmbH, Germany)	95.12% *S. aureus and* 94.7% *E. coli*	No biocompatibility studies evaluated	(96)
		ROS are produced by the photothermic effect, which raises the temperature to kill bacteria		
PCL/Ag	Extrusion	99% *E. coli*	80% cell viability hFOB human osteoblast	(25)
		ROS Induction breaks the cell membrane cell membranes	No *in vivo* study	
TCP/Sodium Alginate (SA) with Ag	Rapid Prototyping (The Fab@home printing model)	99% *S. aureus*	120% cell viability. Osteoblast cell’s full information not provided	(27)
		ROS Induction breaks the cell membrane cell membranes	No *in vivo* study	
PCLA/HA doped with green tea epigallocatechin-3-gallate	FDM followed by coating.	97% *S. aureus*	110% cell viability MC3T3-E1	(97)
			No *in vivo* study	
PCL/Mg	3D FDM microfabrication machine (FDM 3000 System; Stratasys Inc.)	Not studied	No toxicity found in Rat bone marrow stromal cells (rBMSCs)	(34)
PLGA/Mg	Low-temperature rapid-prototyping technique	Not studied	20% MC3T3-E1 cells	(98)
			No *in vivo* study	
PLGA/TCP/Mg	Rapid prototyping (RP) technology	99% *S. aureus*	Relative proliferation rate with MC3T3-E1 cells.	(99)
			No *in vivo* study	

Selecting the appropriate *in vitro* antimicrobial testing could be challenging, given the vast number of available agents and types of materials. The selection of the testing methodology may be based on the following factors:

- Flexibility
- Reproducibility
- Ease of performance
- Accuracy
- Cost-effective

There is a compelling need to opt for a testing methodology that is accurate, cost-effective, and, more importantly, reproducible. The reproducibility of the methodology depends on the efficacy of the antimicrobial agent being tested for each microbe and the microbial environment. Here, a significant task of the clinical microbiology laboratory is the performance of antibacterial susceptibility testing of essential bacterial isolates. The main aim of testing is to detect multidrug resistance in common pathogens and to ensure the susceptibility to antibacterial activities of a particular choice of infections.

Based on the available literature, to assess the antimicrobial properties of materials, three assays are predominantly used: (1) Agar Diffusion, (2) Dilution inoculation, and (3) Contact method. The other methods used for determining the antibacterial behavior are the Touch-transfer inoculation assay/Time-kill test, which is also a potential technique for establishing the bactericidal effect on scaffolds. It is a tool for determining the interaction between the microbial stain and antibacterial agent and further obtaining the information. The time-kill test shows the time-dependent and concentration-dependent antibacterial effect. The other one is the swab inoculation assay, which was based on a touch-transfer inoculation assay for obtaining reproducibility. It also assesses the survival capability of bacteria on the film surface.

## 4 3D Bioprinted *In Vitro* Models for Bacteria

Through cell culture tests, a significant understanding of insights into host–pathogen interactions and infection preclinical mechanisms. However, the *in vitro* bone tissue engineering models are limited to primary characteristics present in the actual physiological environment, pathogen interactions, and infection regulation. 3D cell culture techniques such as biodegradable polymeric scaffolds, and programmable and personalized platforms to engineer cell-laden/scaffolds/constructs have been under evolution to imitate host tissues. The following benefits can be obtained through the development of the customized 3D-printed tissue system.

(i) The modeling of host-bacterial interactions during *in vitro* environment
(ii) Testing of antibacterial behavior of 3D tissue constructs
(iii) Testing under the formation of biofilm

Generally, bacteria grow within 3D structured inhabitants, which form due to multiple species of bacteria found in the human body. The aggregations of the bacterial population play a key role in the communication and characteristics of the community. Geometry affects the viability and pathogenicity of bacteria. 3D printing of bacterial populations gives a chemically interactive, physical arrangement with a suitable density, shape, and size. For bone tissue engineering, there are various methods for creating new platforms for scaffolds using live bacteria, functionalized scaffolds preventing infections, and bacterial-produced scaffolds.

The novel approaches use live bacteria as the porogen, which can sacrifice for decellularization for creating patterns in a 3D bioprinted scaffold, which can broadly be applied to bone tissue engineering applications.^100^ Miniature drug-screening platforms were used in bioprinting that evaluates the biochemical reactions in a picoliter-scale volume at a fast rate to stimulate the drugs/antibiotics.

## 5 Functional Properties of 3D-Printed Scaffolds

Ideally, in orthopedics, antibacterial scaffolds should be designed by considering other following functional properties such as (i) suitable mechanical behavior, (ii) moderate degradation behavior, and (iii) excellent biocompatibility/bioactivity characteristics within the human body, which are significant for orthopedics. Considering the limitations of each fabrication technology, the focus has been given to the performance of the mechanical properties of the material critical in bone tissue engineering and provided a brief rationale for selecting the materials. The functional properties of 3D-printed scaffolds for orthopedics and a summary of the various fabrication routes is provided.

### 5.1 Mechanical Properties

The segments of bone defects under load require the use of 3D porous scaffolds that can maintain their mechanical integrity during healing. Although biopolymers are potential candidates, they face inadequate mechanical behavior. In this direction, pore structure, the geometry of pores, adjusting printer parameters, and the addition of second phases are tailored to improve the mechanical behavior of 3D-printed scaffolds. The incorporation of a metal as a filler has been promising for enhancing mechanical properties.

Recently, Fe and stainless steel (316L) metal powder were added to PLA to fabricate the 3D scaffolds through the FDM process.^29^ The compressive and flexural strength improvement was realized due to the strengthening effect produced by the metal powder. During printing, residual stress developed due to thermal expansion could be detrimental, leading to scaffold failure, as they can behave as crack initiation points. The scaffolds displayed superior compressive strength due to lower shrinkage, enhancing the bonding capability between the scaffold struts. In another study, the incorporation of HA in PLA scaffolds could not improve the compressive strength because of the higher porosity (the compressive strength of pure PLA 240 MPa and HA/PLA with 1.4 MPa).^101^

Additionally, it has been claimed that adding Cu, bronze, and Ag to the PLA matrix increases the strength of 3D-printed scaffolds.^30^ Due to the effect of reinforcement, the elastic modulus for PLA incorporated with Cu, Ag, and bronze, respectively, increased from 1.54 GPa to 1.65, 1.59, and 1.70 GPa. The elastic modulus of 3D-printed PCL and PCL/Ag were assessed using uniaxial tensile test.^25^ The Young’s modulus of PCL was found to be 0.35 GPa, whereas the Ag incorporated increased it to 1 GPa. The improvement in the young modulus was found due to the inclusion of Ag particles, which acted as fillers, restricting mobility and improving the stiffness. With an increase in the Ag concentration, the mechanical characteristics are also enhanced. Commonly, fillers in the polymer matrix restrict the movement of polymer chains and enhance the rigidity of the polymer scaffolds. However, only two concentrations of Ag were investigated in this study. It is essential to know the best-optimized Ag concentration.

Mg is another metal that can cause a strengthening effect. The ultimate compressive strength and elastic modulus of the 3D-printed porous scaffold were improved by 114.5% and 85.7%, respectively, by adding Mg particles (5 wt %) to the PLLA matrix.^102^ Despite the better mechanical performance, the addition of Mg after 7 wt % is detrimental to the mechanical behavior due to the agglomeration of Mg particles in PLLA/Mg scaffolds produced by the SLS. In another study,^34^ 3 wt % of Mg was added to PCL, which showed superior compressive strength in comparison to pure PCL 3D scaffold because of the strengthening effect of Mg metal fillers in the PCL polymer matrix. The scaffold exhibited mechanical behavior closer to human cancellous bone, which would reduce the impact of stress shielding. Similarly, another report showed an improvement of 134.2% in compressive modulus by incorporating 20 wt % of Mg in the PLGA matrix, which is attributed to higher load-bearing capacity.^98^

A study conducted on the functionalization of PLA with Minocycline (MH) and collagen (Col) had no impact on the scaffolds’ compressive strength fabricated via FDM.^64^ PLA, PLA-Col, PLA-Col-MH, and PLA-Col-MH -HA had strengths of 13.3 ± 2.0 MPa, 12.7 ± 1.9 MPa, 11.2 ± 1.7 MPa, and 13.6 ± 2.0 MPa, respectively. Further, a higher young modulus with a lower plasticity was observed. Table 2 represents the key findings of state-of-the-art 3D-printed scaffolds about the synthesis method, percentage of porosity, size of the pore, and mechanical behavior (strength and elastic modulus).

**Table 2:** Key Findings of State-of-the-Art 3D-Printed Scaffolds in Relation to Synthesis Method, Percentage of Porosity, Size of the Pore, and Mechanical Behavior (Strength and Elastic Modulus)

Scaffolds	Manufacturing Process	Porosity (%)/Pore Size (μm)	Strength (MPa) %	Elastic Modulus (MPa) %	Ref
PLA	FDM	800–920	27.6	0.4	(103)
PLA-10Fe	FDM	820	53.3	10	(29)
PLA-5Ti	FDM	47	45.6	80	(103)
		787			
PLA-Cu	FDM	50	40	4.9	(30)
		400			
PLA–bronze	FDM	50	65.5	27.4	(30)
		400			
PLA-Ag	FDM	50	103.5	13.4	(30)
		400			
PCL-5Mg	FDM	66	50	60	(34)
		480			
PCL-Mg^–^	FDM	400	Not studied	Not studied	(33)
PLGA-15 Mg	Low-temperature deposition manufacturing (LDM)	414 (macropore)	59.8	155	(104)
PLGA-20Mg	LDM	50	134	Not mentioned	(98)
		400			
PLLA-5Mg	SLS	500	114.5	85.7	(102)
		29			

### 5.2 Degradation Behavior of 3D-Printed Scaffolds

The degradation behavior of biodegradable 3D-printed scaffolds in the simulated physiological environment is significant for bone tissue engineering. The *in vivo* bonding of the scaffolds is related to the calcification ability *in vitro*. The scaffold must support an apatite layer formation upon implantation when the surface is exposed to a physiological environment. The formation of apatite layers on the surface of the scaffold helps with bone mineralization. In general, the kinetics of the antibiotic release profile from 3D scaffolds is essentially driven by the degradation, dissolution, and desorption of the surfaces of the scaffolds.

Degradation of scaffolds starts with a series of chemical reactions at the exposed surface in the physiological environment and the formation of an elongated layer upon implantation. This bioactivity is essential for enhancing the bond with natural bone. Additionally, for biodegradable 3D-printed scaffolds, the degradation behavior of the scaffolds must coincide with the rate of bone healing. Hence, it is of utmost significance for the biodegradable scaffolds to undergo a degradation test to ensure that the materials of the scaffolds are removed without any additional surgery after the healing of bone tissue. The biodegradable polymers such as PCL, PLA, and PLGA degrade in the simulated physiological environment because of the hydrolytic biodegradation route/pathway (polymer chains get divided into water-soluble oligomers and monomers).^105^ Nevertheless, it is a long process for the complete degradation of polymers in the human body. The slow rate of deterioration of polymers is one of the difficulties in biodegradable polymer-based scaffolds. Several approaches have been used to maintain the rate of biodegradation of scaffolds without compromising the mechanical property, antibacterial performance, and biocompatibility such as blending, copolymerization, surface modification, and incorporation of metal powders.

In one of the studies, an *in vitro* enzymatic degradation study was conducted to analyze the stable behavior of 3D-printed PCL/Ag scaffolds. Lipase solution was introduced as the medium due to PCL possessing an ester bond which is prone to hydrolytic degradation by lipase. The degradation of the preweighed PCL scaffolds was assessed after they were submerged in a lipase solution. Initially, the degradation of all the scaffolds was less than 10% in 24 h; gradually, the PCL scaffolds reached 82% after 20 days. As mentioned, surface degradation happens for polymers by direct contact with the polymer and enzyme.^106^ Scaffolds with interconnected pores and a large surface area facilitate the degradation of enzymes. The concentration of the enzymes adsorbed on PCL determines the degradability of scaffolds and the polymer ester chains exposed to enzymes. On the other hand, adding Ag changes the spatial distribution of PCL scaffolds, decreasing the adsorbed lipase enzymes and ester polymer chains, which results in the degradation of PCL/Ag scaffolds.

Compounding with metal powder has been an effective technique due to its ability to concurrently increase the mechanical strength and biodegradation rate. It has been reported that the biodegradation rate of PLA porous scaffolds was amplified with the 10% vol incorporation of iron.^29^ The phosphate-buffer saline (PBS) was used as an immersion medium for soaking PLA/Fe scaffolds. The formation of hydroxide (OH^–^) and iron oxide was observed, which can easily be removed *via* urine excretion (eq 1). The pH level also increased along with the weight of the scaffold, which signifies a high corrosion rate of the PLA/Fe porous scaffold, i.e., 0.18 mm/year in comparison to pure PLA scaffolds (0.01 mm/year). After immersion, the samples were delaminated and swelled, leading to the degradation of mechanical behavior. This was attributed to hydrolysis, voids, and defects, which allowed infiltration of the PBS solution and resulted in weak layers of 3D-printed porous scaffolds. On the other hand, Fe has a good Pilling-Bedworth ratio, so iron oxide formation prevented the surface of the porous PLA/Fe scaffold from further propagation of biodegradation behavior while maintaining mechanical integrity:
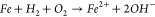
1

Wettability is another factor influencing the biodegradation rate of the porous 3D porous scaffolds. It has been pointed out that the decrease of water contact angle (WCA) from 95.4 to 70° of pure 3D-printed PLA and PLA/Fe scaffold is another reason behind the enhanced degradation rate. Another study showed that adding 5 wt.% of Mg into the polymer PCL matrix also modified the WCA of PCL from 98.2° to 59.0°, resulting in a hydrophilic surface favorable for degradation.^34^

**Figure 11:**
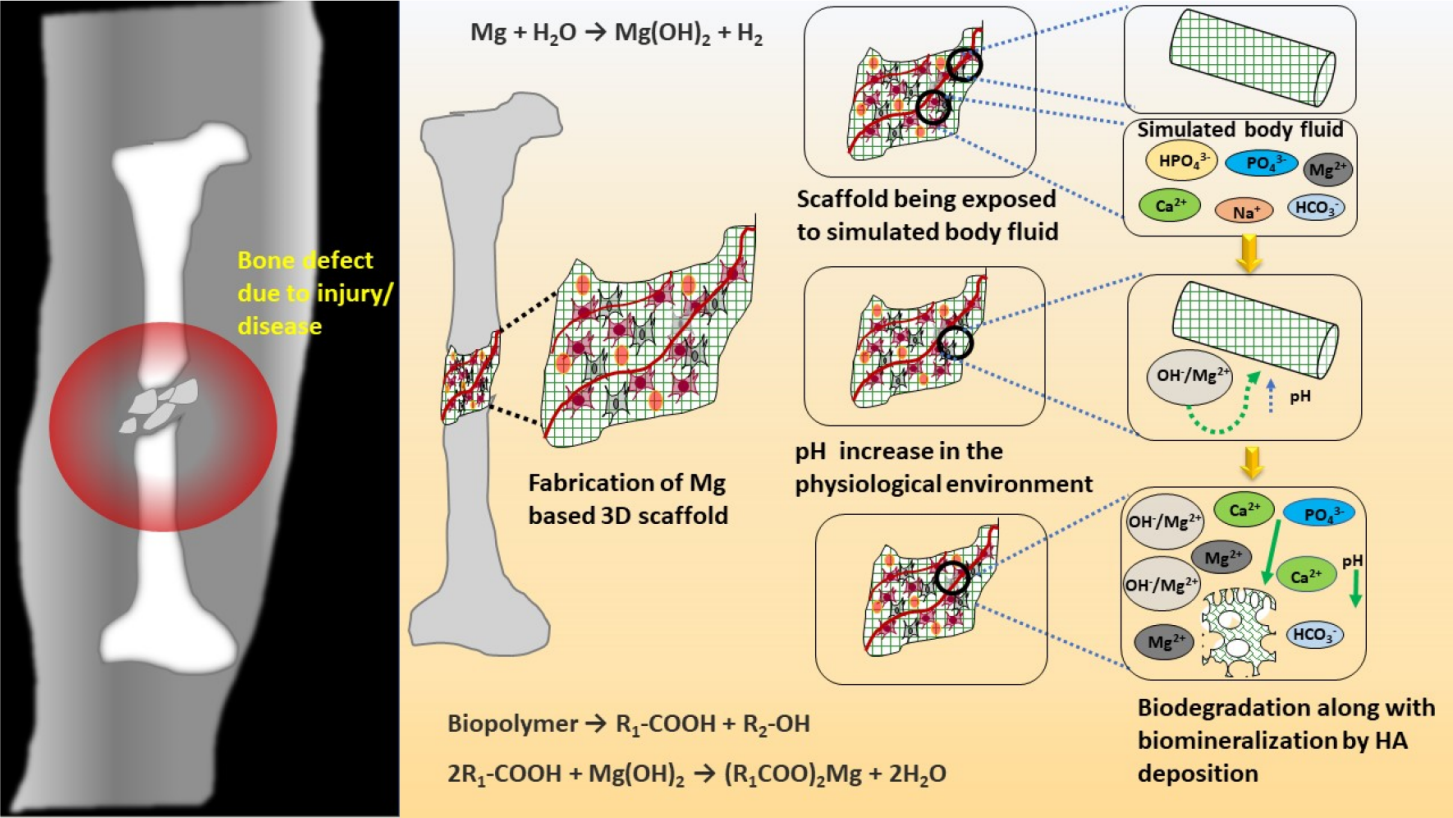
Schematic of the biocorrosion behavior of 3D-printed Mg/PCL scaffolds. Body fluid is a complex environment containing several ions and biomicro and macromolecules, which directly or indirectly affect the degradation behavior of bone substitutes in the body. The degradation of scaffold materials (i.e., Mg and PCL) surrounding the body fluid increases the pH due to the formation of oxide groups. Along with the degradation, the biomineral deposition starts at the scaffold surfaces (due to the reaction of ions from body fluid and scaffold), which eventually helps in the adsorption of protein followed by cell adhesion and proliferation. In addition, the deposition of the biomineral layer at scaffold surfaces enhances the degradation resistance by providing temporary support (R_1_ and R_2_ denote the different polymer chains depending on the number of carbon present).

The incorporation of Mg fillers into PCL, PLA, and PLGA^102,34,104^ polymer matrix through the 3D printing approach has accelerated the biodegradation behavior of the 3D scaffolds *via* the following mechanisms: (i) Penetration of chlorinated media through the interface of Mg/polymer due to the superhydrophilic nature of the scaffold surface (ii) Mg(OH)_2_ formation (eq 2) (iii) biodegradable polymer hydrolysis and ester bonds formation as the acidic corrosion products (eqs 3 and 4). The corroded product of Mg (i.e., alkaline) and biodegradable polymer (acidic) are consumed by each other (eq 4), which promotes the biodegradation of Mg and polymer. Therefore, Mg plays a remarkable role in tailoring the slow degradation of biodegradable polymers for the bone regeneration process.^107^Figure 11 illustrates the degradation process of the Mg/PCL scaffolds:
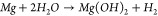
2
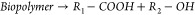
3
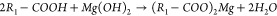
4

R_1_ and R_2_ denote the different polymer chains depending on the number of carbons in the polymeric chains.

### 5.3 Biocompatibility of 3D-Printed Scaffolds

The key characteristics of bone tissue engineering scaffolds involve suitable cell attachment, spreading proliferation, differentiation, no toxic byproducts, microbial resistance, and noninflammatory response for clinical applications.^108,109^ Biodegradable polymers are the most biocompatible materials with negligible toxic byproducts available in bone tissue engineering applications. The osteoconductivity of the 3D-printed scaffolds can be enhanced by adopting the suitable porous-construct-based design of the scaffold. Researchers have introduced the index of bioactivity based on the amount of time needed for bioactive materials to bond between human bone and synthetic bioactive scaffolds. Even the architecture of the pore served as a useful tool for optimizing bone-scaffold properties specifically for patient needs. Osteoconduction and bone grafting require a variety of pore diameters. PCL was coated over a TCP scaffold to improve osteoconduction.^110^ The *in vivo* data showed that releasing the drug alendronate from the PCL-coated scaffolds promotes bone formation. Alendronate is an essential drug used for treating osteoporosis.

The drug delivery and growth factors to the 3D-printed scaffolds during bone mineralization are essential. The process of *in vivo* bone healing has been dramatically improved by incorporating vancomycin and rifampin into TCP-based scaffolds. After 28 days, the distal and primary ROIs had fully new and resorbed bone. In another study, the cytocompatibility of PLGA and PLGA/β-TCP scaffolds demonstrated profound cell adhesion, proliferation, and growth. The results showed that despite β-TCP’s high osteogenic potential, its involvement in cell proliferation or differentiation had no appreciable impact.^111^ Cell viability demonstrated that after 24 h of implantation, the mesenchymal stem cells adhered to the surface and began to release protein, alkaline phosphates, and collagen, which induces osteoconduction of the scaffolds.

To further enhance the biocompatibility and microbial resistance activity of 3D-printed biodegradable polymer scaffolds, it is accomplished by incorporating metal fillers.

One study evaluated the cytocompatibility behavior of pure PLA, PLA/Fe, and PLA/stainless steel (316L) fabricated through material extrusion.^29^ PLA/Fe composite scaffolds showed better cell proliferation and differentiation compared to those of pure PLA and PLA/316L scaffolds. The improvement is ascribed to the hydrophilic nature of the PLA/Fe porous scaffold, a very favorable condition for cell adhesion.

Adding Mg fillers into biodegradable polymers like PCL, and PLA through the 3D printing method has a remarkable impact on the cytocompatibility of 3D porous bone scaffolds. Mg has hydrophilic nature, which is beneficial to cell attachment, proliferation, and differentiation; it creates bioactive sites on its surface and moderate alkaline pH ≤ 10, a preferential conducive microenvironment for the growth of cells.^33^ In contrast, a rise in pH level of more than 10 is detrimental for cells; hence, optimizing the weight percentage of Mg in the biodegradable polymer is crucial. It has been presented that the use of Mg beyond 5 wt % in PCL and 10 wt.% in PLGA porous scaffolds severely impacted cytocompatibility because of the raised pH as a result of an accumulation of Mg^2+^ in the cell culture media.^34,104^

PLGA-Mg scaffold was designed using a low-temperature rapid prototyping (LT-RP) technology. Mg was incorporated in PLGA scaffolds aiming for its versatility as an all-in-one platform for postsurgical osteosarcoma (OS) management. The *in vitro* antitumor behavior of the PLGA-Mg scaffolds is evaluated on osteosarcoma Saos-2 cells. MC3T3-E1 cells are used to assess the outcome of the PLGA-Mg scaffolds in promoting osteoblasts-like cell adhesion, differentiating, and bone mineralization. Besides, *in vivo* osteosarcoma surgical intervention was performed by using a mouse model to determine the antitumor efficacy. Osteogenic effects were evaluated by the bone defect model at the distal femur of a rat. The PLGA with 10 wt % Mg (P10M) showed better osteogenic properties compared to others. P10 M scaffolds were successfully used in *in vivo* to cure postsurgical osteosarcoma (OS) and bone regeneration to show that they could easily produce a photothermal effect at the implantation site when exposed to an 808 nm NIR laser, eliminating any remaining Saos-2 cells and completely suppressing OS recurrence suppression and bone regeneration.

#### 5.3.1 Osteogenesis Properties of 3D-Printed Scaffolds

It is well-known that bones are an essential category of connective tissue found in vertebrates as they deliver a framework to the body, a particular shape, protecting the internal organs and the foundation of movement and locomotion.^112^ The phenomenon of bone formation is called osteogenesis, and their calcification is termed ossification. The categories of osteogenic materials involve biodegradable and nonbiodegradable polymers; unique glass ceramics described as bioactive glasses (BG); calcium phosphates (Ca, HA; TCP; and biphasic calcium phosphate (BCP)); and biomaterials derived from natural sources (e.g., bovine or human bone or corals).^113^ The standard mechanism involved here is bioceramics dissolving in the body, seeding with new bone formation, and releasing Ca and P ions into the biological medium. The transformation and dissolution processes of HA with a particular microstructure, likewise the micropore, spaces existing between the single crystals into the grains of the bioceramics and macrostructures with mesopores and macropores represent a physicochemical process, proteins-crystal interactions, colonization of cells and tissue, remodeling of bone, and finally contributing to ingrowth of the bone. The biological precipitation of apatite and dissolution of Ca–P must occur simultaneously with bone formation and osteoid after implantation in bony and nonbony sites.

The incorporation of metals shows a promising method to enhance the osteogenic ability of nonbioactive polymers.^114^ Incorporating Mg into the monophase PCL and PLGA bone scaffolds implies positive effects as it has promoted the osteoblast gene expression ability and significantly promoted mineralization.^34,104^ Mg as a filler material was helpful in expressing neuronal calcitonin gene-related polypeptide-α (CGRP), implying robust bone formation. The *in vivo* analysis through histology showed that the pure PCL and PCL with 3 wt % of Mg showed the formation of new bone tissue for a bone defect.^34^ Adding Mg to the PCL scaffold exhibits efficient bone formability in comparison to pure PCL scaffolds as more dense collagen fibers are generated as per Hematoxylin and Eosin (HE) and Masson staining. Through the femur defect rat model, the micro-CT scans show the formation of new cortical and cancellous bone with 10 wt % of Mg into PLGA scaffold fabricated through low-temperature deposition manufacturing (LDM), which confirms the tremendous potential of the Mg-based scaffold for bone tissue engineering.

Blood takes care of the basic supply of nutrients and oxygen to the tissues and organs along with the discretion of their waste metabolites. In this connection, the vascular networks must be established within the scaffold, while bone healing is significant. The clinical transformation is greatly affected by the inadequacy of angiogenesis in bone tissue engineering.^115,116^ Vascular endothelial growth factors (VEGF) were investigated to analyze the efficacy of pure PCL and PCL with Mg porous scaffolds through enzyme-linked immunosorbent assay (ELISA) and real-time quantitative polymerase chain reaction (RT-qPCR) method. Mg/PCL biodegradable scaffold fabricated via FDM technique showed superior protein expression and VEGF response compared to those without Mg filler. The influence was due to Mg^2+^ ions activating the extracellular matrix protein and transcription factors associated with bone reconstruction.

The biological performance of the 3D-printed scaffolds in orthopedics can be improved by implementing strategies like coating. Coatings produce a nanostructured surface on the scaffold with numerous micropores (size <100 μm) conducive to cell attachment. Drugs can be added to the coatings and promote the biocompatibility of the 3D scaffolds. It has been reported that adding a small concentration of metallic fillers can enhance the osteogenic property of the 3D scaffolds.

Mg-based scaffolds with possible antibacterial properties need to be critically investigated. In an effort, calcitonin gene-related polypeptide-α (CGRP) involved a mechanism that focuses on the osteogenic effects of Mg-based implants.^117^ An innovative intramedullary nail (IMN) system was developed, and it showed potential for healing bone injuries for low-energy osteoporotic fractures. It might be able to deliver Mg^2+^ ions or recombinant CGRP to certain healing regions in the bone by developing cell or tissue targeting systems. The findings from both *in vitro* and *in vivo* experiments support the role of Mg in the substantial increase of bone formation.^117^ The benefits of Mg are primarily mediated by the calcitonin gene-related polypeptide (CGRP), a neuropeptide, which is released by sensory neural endings in the long bone shaft. Although the importance of the central nervous system in controlling bone development is well-known, the precise function of sensory neurons in this process is yet unknown. The major underlying mechanism is the CGRP-mediated cross-talk pathway between peripheral nerves and periosteum-derived stem cells PDSCs.

Researchers have also even examined the synergetic effects of antibacterial and osteogenic behavior. In this direction, a study was conducted to construct the balance between antibacterial and osteoblast functions, which was varied by controlling the content and composition of Ag@GO nanocomposites in the scaffolds.^44^ The bacterial membrane may be damaged by silver, which may also inhibit the metabolic process of some enzymes by binding with thiol (sulfhydryl) groups in proteins and enzymes. The concentration of Ag is the deciding factor in osteoblastic differentiation. The release of Ag ions from the scaffolds was controlled well. During the short period, Ag might hinder cell proliferation, but it is accepted due to the long process of bone construction promoting osteoblast cell differentiation.

In addition to the engineering and manufacturing aspects mentioned above, knowledge gaps and uncertainties exist, for example, the contribution of nourishing factors, such as various cell types, growth factors, how vascularization is evolving over time, cell survival, and maturation within the prefabricated constructs. Another major unanswered question is whether bioprinting can lead to better cell seeding and implantation.

#### 5.3.2 Biosafety of Scaffolds

The biosafety of the scaffold material is another important criterion when implanted directly into the body. The series of standard evaluations for medical biomaterials and devices are the methods to which biosafety assessments are commonly referred to. The 3D-printed scaffolds may deteriorate and segregate free particles during movement after being implanted in bone defects, which could result in embolism or inflammation. Day-to-day physiological activities are affected by this phenomenon. The Regenovo 3D Bio-Printer (Regenovo Biotechnology, China) was used to prepare a hybrid HA/carboxymethyl chitosan/polydopamine scaffolds. Biochemical tests were performed to determine the Ca ions in serum and other blood biochemical markers. It was observed that the Ca and inorganic P level was found to be normal after 12 weeks of the implantation.^118^ Another study revealed the release of Mg ions from a low-temperature deposition 3D printing machine (CLRF-2000-II, Tsinghua University, Beijing, China). Mg ion level in serum (from 0 to 12 weeks) and biochemical blood indicators after the surgery were analyzed. Liver function (indicators of alanine aminotransferase (ALT), aspartate transaminase (AST), albumin (ALB), globulin (GLB)), and kidney function tests (indicators of creatinine (Cr), and blood urea nitrogen (BUN)) showed that indicator levels are in the normal range in all scaffolds.^119^ Further, immune response indicators (such as immunoglobulin G (IgG) and immunoglobulin M (IgM) tests) showed that no significantly different levels of Mg ions release in blood circulation, which induced no immune responses. Despite that, the higher level of Mg ion concentration will be a concern for biosafety due to potentially increased risk of liver disease, cardiovascular, and metabolism disorder.

## 6 Commercialization of 3D-Printed Scaffolds

Globally the market size of scaffolds was valued at ∼1.1 million USD in 2020, which is expected to expand with a compound annual growth rate (CAGR) of 8.4% from 2021 to 2028.^120^ Such drastic market growth is primarily attributed to the demand for 3D-printed scaffolds for bone defects and translation research. The rapid shift in the paradigm of bone defects is to tailor the challenges that are correlated with the drug development process, which is anticipated to catapult the growth of the market for scaffold technology.

The use of 3D scaffolds for bone defects is growing, as they can efficiently mimic the structure of natural bone. Thus, 3D-printed bone scaffolds have emerged as an innovative technique for the early discovery and therapeutic solutions for infected bone defects. Additionally, recent technological advancements in this field led to the escalating adoption of 3D bioprinting for bone tissue reconstruction procedures. 3D bioprinting technology can be considered one of the major profitable advancements in bone tissue engineering, where cell-laden biomaterials can be directly printed to form scaffolds. Several 3D bioprinted scaffolds have been developed to tailor the functional properties for bone tissue regeneration. In November 2020, CELLINK GLOBAL took the initiative to enter the TRIANKLE consortium to establish a gelatin and collagen-based scaffold *via* 3D printing technology for application in joint tissue.

Other companies, such as Osteopore International, include Osteomesh, Osteoplug, and Osteoplug-C in various sizes, and also customized implants are fabricated based on patients’ and surgeons’ treatment plans. TRUMATCH CMF Titanium 3D-printed Plates and Guides is another 3D-printed product by DePuy Synthes, a direct and fully guided material system. Another Fortune 500 Michigan-based medical device manufacturer, Stryker, developed six additively manufactured porous implants.

Besides, the COVID-19 pandemic acted as a positive catalyst for the antimicrobial 3D-printed scaffold technology market. The application of bone tissue engineering is used extensively for understanding bacterial infection, developing the *in vitro* model system, and finding suitable therapeutic solutions to deal with bacterial infections.

## 7 Challenges and Prospects

The requirement for innovative strategies for healing bone defects and infection in an ever-increasing population is self-evident. Despite the development of state-of-the-art fabrication techniques and innovative biomaterials for bone tissue engineering, very few technologies and biomaterials are reaching clinical evaluation for the most basic of structural biocompatibility issues. It might not be wrong to say that this discipline is still in its infancy. Several unmet challenges and limitations need to be overcome for further continuation of research and clinical translation of biomaterials. However, biomaterials fabricated through 3D printing technologies are receiving broad endorsement for justified functional properties in terms of antibacterial, mechanical, and biocompatibility properties. Although antibacterial 3D-printed scaffolds have created a revolutionary decade in bone tissue engineering, they still face many challenges in different aspects that need to be tailored for the innovation of medical devices. The complex and customized shapes are still a bigger challenge for 3D bioprinters.^121^ Regarding all present substantial vascularization structures, integration with host tissue, and, to date, economic viability, the usual challenges remain. In addition, the recent technological developments in the bone tissue engineering field create an exciting environment enabling further opportunities, but there is still a lack of clinical application of experimental results.

### 7.1 Antibiotics-Induced Antibacterial Strategy

The major issue is drug resistance to bacteria and can be called drug abuse. Hence, increasing the efficiency of the antibacterial capability of scaffolds while avoiding drug abuse is of prime importance. Designing and improving the smart response of scaffold materials, such as controlled localized drug release to the infected area, might be a potential solution.

### 7.2 Ion-Mediated Antibacterial Strategy

It achieves favorable antibacterial behavior owing to the ability of sustained the release of metal ions. Generally, the metal ions involved in this method are often relatively heavy metals (Zn, Mg, Au, and Ag); they face risks biologically and are a barrier in clinical applications. Hence, it is vital to balance the antibacterial ability and biocompatibility. The antibacterial metal ions should possess some essential properties, such as osteogenesis, immunoregulation, and angiogenesis. The effect of the type and composition of metal ions concerning the antibacterial assessment, along with all of its influencing factors, should be considered for achieving the optimum outcome.

### 7.3 Coating Strategy on the Scaffolds

The stability and durability of the antibacterial coatings on scaffolds are the major limitations, as they should not be confined within a few hours. The adhesion of the coating with scaffolds is another challenge that needs to be tailored by understanding the chemical characteristics of the coating materials and the intended surfaces. So, finding a suitable coating is another essential challenge that needs to be taken.

### 7.4 Sequential Antibacterial Behavior and Osteogenesis of Scaffolds

Another critical parameter that needed to be considered and missing in the literature is the time taken by bone healing that coincides with the antibacterial activity. Visualizing the combined effect of the antibacterial and osteogenesis is another essential criterion to be focused upon. There are studies on Ag@GO nanocomposites to control antibacterial activity along with osteogenesis.^44^ Ag can reduce the bacterial growth along with promoting the osteoblast cells. Thus, using cutting-edge printing technologies, it is necessary at this point to strike a balance between osteoblast functions and antibacterial activity.

### 7.5 Importance of Manufacturing Parameters/Methods

To overcome the challenges, the combination of several approaches with their synergy could be used effectively while enhancing the antibacterial effect. Another major challenge is to complete the design and fabrication of the 3D scaffolds without compromising the osteogenic properties. Although 3D printing still has several advantages, some challenges need to be solved in the near future: (1) Bone is a natural tissue with a hierarchical structure; therefore, 3D-printed scaffolds should mimic the same structure. However, most 3D-printed scaffolds through extrusion have limited resolution, which causes clotting in the nozzle. Therefore, there is a need for an advanced nozzle with a significantly higher resolution. (2) Infected bone defects show a heterogeneous structure with mechanical gradient properties, so producing complex structures with gradient functional properties, is challenging to mimic the ECM structure. (3) It is essential to disinfect the infected area with the scaffolds along with excellent vascularization for providing sufficient transport of nutrients or oxygen during the regeneration of bone. Thus, sufficient release of antibacterial agents and angiogenic agents and generation of the vascular-like channel in 3D-printed scaffolds are needed.

Currently, available 3D printing technology, including 3D bioprinting, is the only one that produces cell-incorporated scaffold structure; no other 3D printing technique enables cell incorporation during the printing process. Hence, the need of the hour is to invent 3D printing techniques that have the dual function of scaffold synthesis and simultaneously cell incorporation. Hence, 3D-printed scaffolds with excellent ability for treating infected bone defects are needed.

4D and 5D printing using time as an entity could be a beneficial tool for creating complicated structures on demand controlling shape and functions, considered the next generation for bone tissue engineering. This technology provides the possibility for personalized treatment and precision medicine, which is seen as a paramount concern in bone tissue engineering. Indeed, stimuli-responsive biomaterials and novel strategies have been developed, but 4D bioprinting still faces many challenges that need to be addressed.

It is very challenging to produce printable stimuli-responsive scaffolds and further transform them into bioinks. Further, shape transformation through 4D printing (such as folding or assembling) still does not meet the complex needs of clinical applications. There is a need for more efforts to achieve accuracy in shape-transformation for printing in bone tissue engineering. Controlling and releasing internal stress precisely is another challenge while using stimuli-responsive materials. The scaffold should not lose its unique properties while maintaining stimuli-responsive capabilities for the long-term application process.

In addition, the human physiological environment is complex, consisting of cellular activity, and might get influenced by various stimuli (e.g., neuro regulation, self-regulation, and humoral regulation). 4D-printed biological constructs undergo many transformation processes before they achieve full functionality. Hence, it is very challenging to print a scaffold that undergoes a shape transformation along with multiple stimuli and functional transitions simultaneously.

Moreover, orthopedic applications of scaffolds demand complex and robust structures with curved surfaces. 5D printing stands out in printing curvature structures with high strength and offers printing of the structure as per the actual surgery of the patient. The development of suitable 5D printing technology might offer unlimited possibilities and provide excellent support to save the life and time of the patient. It has the capability of printing complex-shaped scaffolds to fulfill the immediate requirements of bone tissue engineering.

## 8 Summary and Outlook

3D-printed scaffolds with high antibacterial behavior, degradability, mechanical property, bioactivity, and osteogenesis are significantly beneficial to cure bone infections and defects. 3D-printed interconnected porous scaffolds facilitate cell nutrient transportation and migration while releasing the active substances, promoting osteogenesis and angiogenesis, ultimately repairing the bone. FDM, LDM, and SLS techniques have been most commonly used to 3D print the scaffolds. Various materials are mixed initially and then printed in layer-by-layer fashions in these techniques. Further, the requirement of prescribed shape and functionality for the infected bones can be attained by the application of 4D and 5D printing with the help of external or internal stimuli. These techniques produce complex shapes and smart scaffolds, which might provide conciseness during the *in vivo* implantation. For sustainability and durability of the 3D-printed scaffolds in the physiological environment, the scaffold structure should possess the optimum mechanical, biodegradation, and biocompatible properties. The mechanical strength of scaffolds manufactured by various 3D printing techniques depends on the physical properties (e.g., porosity, large pore size) and the addition of the secondary fillers. Also, adding metallic elements can improve the biodegradation resistance of polymeric scaffolds. These secondary fillers affect the swelling and delamination along with the potential of layer intersection during soaking experiments. Even the hydrophilic/hydrophobic nature of 3D-printed scaffolds is affected by the incorporation of metallic particles/filler, which alters the biodegradation behavior.

Scaffolds fabricated through 3D printing technology to provide antibacterial behavior are necessary to circumvent bacterial infections, which dramatically affect the implanted scaffold’s success. The antibacterial property of the scaffolds against bacteria and the formation of biofilm can be obtained by merging the scaffold materials broadly with antibacterial agents like antibiotics, antibacterial polymers/peptides, metals, glass, bioceramics, carbon materials, composites, and combined strategies. The number of drug-resistant bacteria is increasing alarmingly; alternative regenerative medical models are necessary for ensuring safe clinical treatments. The state-of-the-art 3D-printed antibacterial scaffolds used to impede microbial infections in bone tissue engineering have been critically evaluated in this study. Nevertheless, the antibacterial mechanisms involved in infected bone defects, which can impede bacterial infections and the formation of biofilms, require additional investigation. In the majority of studies to secure clinical transfer, the toxicological features of these antibacterial scaffolds have been the main focus. The toxicological aspects of these antibacterial scaffolds have been emphasized in most research for safe clinical transfer. The *in vitro* assessment in terms of cell viability and cell differentiation, along with osteogenic ability, have been mentioned. After the compliance of satisfactory functional properties, 3D-printed scaffolds for bone tissue engineering will imply their potential for clinical application.

The COVID-19 pandemic also has led to a burden on the healthcare system for determining antibacterial strategies against the spread of emerging pathogens. 3D bioprinting of the scaffolds could be an impressive technology for dealing with health issues due to infections. No functional bone–organ construct has been synthesized by using 3D printing technology. Using 4D printing, cell aggregates and organoids emerge in a path that is still in the infant stages for the treatment of large bone defects.
